# Inverse probability weighting-based mediation analysis for microbiome data

**DOI:** 10.1093/bioinformatics/btag750

**Published:** 2026-10-13

**Authors:** Yuexia Zhang, Jian Wang, Jiayi Shen, Jessica Galloway-Peña, Samuel Shelburne, Linbo Wang, Jianhua Hu

**Affiliations:** Department of Statistics and Data Science, The University of Texas at San Antonio, San Antonio, TX 78207, United States; Department of Biostatistics, The University of Texas MD Anderson Cancer Center, Houston, TX 77030, United States; Statistics for Clinical Development – US, BeOne Medicines, San Carlos, CA 94070, United States; Department of Veterinary Pathobiology, Texas A&M University, College Station, TX 77843, United States; Department of Infectious Diseases, Infection Control and Employee Health, The University of Texas MD Anderson Cancer Center, Houston, TX 77030, United States; Department of Statistical Sciences, University of Toronto, Toronto, ON M5G 1Z5, Canada; Department of Biostatistics, Columbia University, New York, NY 10032, United States

## Abstract

**Motivation:**

Mediation analysis is an important tool for elucidating causal mechanisms in biomedical research and has recently gained increasing attention in microbiome studies. Motivated by a microbiome study of acute myeloid leukemia (AML) patients, we investigate whether the effect of induction chemotherapy (IC) intensity on infection status is mediated through gut microbial taxa. This problem poses substantial methodological challenges due to the high dimensionality, zero inflation, and dependence of microbial mediators, as well as the presence of an exposure-induced mediator–outcome confounder, antibiotic use.

**Results:**

To address these challenges, we propose a novel nonparametric identification formula for the interventional indirect effect, a causal mediation estimand that remains identifiable in the presence of exposure-induced mediator–outcome confounding. Building on this identification result, we develop an inverse probability-weighting estimator and construct standard normal bootstrap confidence intervals for inference. Simulation studies demonstrate that the proposed method achieves accurate effect estimation and maintains appropriate type I error rates and power in finite samples. Application to the AML microbiome study provides evidence of a statistically significant interventional indirect effect of IC intensity on infection risk through alterations in the gut microbiome.

**Availability and implementation:**

The R code for the simulation studies is available at https://github.com/YX-ZHANG-1/CausalMediationMicrobiome and archived on Zenodo at https://doi.org/10.5281/zenodo.21960802

## 1 Introduction

The human microbiome has emerged as an important contributor to health and disease, with alterations in microbial composition linked to a wide range of complex conditions, including cardiovascular disease, inflammatory bowel disease, and cancer (Koeth *et al.* 2013, Zackular *et al.* 2016, Halfvarson *et al.* 2017). Advances in next-generation sequencing technologies, such as 16S rRNA and shotgun metagenomic sequencing, have enabled high-resolution characterization of microbial communities and facilitated systematic investigation of the clinical, genetic, and environmental factors shaping the microbiome and its functional impact on human health (Chen and Li 2016, Wang *et al.* 2021). Increasing evidence further suggests that the microbiome may mediate the effects of clinical and dietary factors on health outcomes. For instance, allogeneic hematopoietic stem cell transplantation has been shown to disrupt gut microbial communities, with bacterial domination associated with increased infection risk (Taur *et al.* 2012). Related mediation effects have also been reported for the association between dietary intake and immune responses or chronic diseases (Sivan *et al.* 2015, Koslovsky *et al.* 2020). A deeper understanding of how the microbiome links risk factors to health outcomes may inform novel strategies for disease prevention and intervention through targeted modulation of microbial communities (Faith *et al.* 2013, Le Chatelier *et al.* 2013).

Motivated by a microbiome study of acute myeloid leukemia (AML) patients conducted at The University of Texas MD Anderson Cancer Center (MD Anderson), we investigate whether gut microbiome features mediate the causal effect of induction chemotherapy (IC) intensity on infection risk. Infections in cancer patients frequently originate from commensal bacteria (Montassier *et al.* 2013), linking infection risk closely to alterations in the gut microbiome (Zitvogel *et al.* 2015). Patients with AML receiving intensive IC are particularly vulnerable to microbiota-derived infections (Gardner *et al.* 2008), which contribute to treatment-related morbidity and mortality (Cannas *et al.* 2012). Preliminary analyses of AML patients at MD Anderson further showed that baseline microbiome α-diversity and treatment-induced changes in diversity were associated with infections occurring during and shortly after IC (Galloway-Peña *et al.* 2016, 2020). Together, these findings suggest that alterations in the gut microbiome may mediate the effect of IC intensity on infection risk in patients with AML.

Mediation analysis provides a principled framework for understanding how and why causal effects arise. Classical mediation methods in the social and health sciences are typically formulated within the linear structural equation modeling framework (Baron and Kenny 1986, MacKinnon 2008). Related approaches have recently been adopted to study mediation effects of microbiome features in human health and disease (Zhang *et al.* 2018, 2021). However, mediation effects defined under linear modeling assumptions are model driven and do not readily generalize beyond linear models. In particular, they are not well suited for settings with binary outcomes, such as infection status in our study. In contrast, modern causal mediation analysis relies on nonparametric definitions and identification results. Foundational work by Robins and Greenland (1992) and Pearl (2001) established nonparametric notions of direct and indirect effects and conditions for their identification. Building on this framework, several methods have been developed to address the high-dimensional and compositional nature of microbiome mediators. These include sparse compositional mediation models for continuous and binary outcomes (Sohn and Li 2019, Sohn *et al.* 2022), regularized causal mediation approaches using log-contrast and Dirichlet regression models (Wang *et al.* 2020), and methods tailored to zero-inflated microbial mediators (Liu *et al.* 2025).

Despite these advances, existing microbiome mediation methods cannot be directly applied to our AML study due to the presence of exposure-induced mediator–outcome confounding. In particular, antibiotic use both influences gut microbiome composition and infection risk and is itself affected by IC intensity. Thus, ignoring antibiotic use leaves the microbiome–infection relationship confounded, whereas conventional adjustment is problematic because antibiotic use is itself affected by IC intensity. This structure is common in clinical microbiome studies. To address such settings, VanderWeele *et al.* (2014) introduced the interventional indirect effect, which remains identifiable in the presence of exposure-induced mediator–outcome confounders and proposed a weighting-based estimation strategy. However, their approach requires explicit modeling of mediator distributions, which is challenging in our AML microbiome study where mediators are high-dimensional, zero-inflated, and dependent (Martin *et al.* 2020).

To address this challenge, we derive a novel identification formula for the interventional indirect effect that avoids explicit modeling of the conditional distribution of mediators, thereby eliminating the need to specify a potentially complex high-dimensional model. Instead, the proposed approach only requires modeling the conditional expectation of the binary infection outcome, the conditional distributions of the binary antibiotic-use variable, and the exposure propensity score. To accommodate the high dimensionality of microbial mediators, we incorporate sparsity-inducing regularization when modeling the outcome. In addition, we apply the centered log-ratio (clr) transformation to account for the compositional nature of microbiome data (Aitchison 1982, Lin and Peddada 2020). The presence of the interventional indirect effect is assessed using a standard normal bootstrap confidence interval (CI) (Efron and Tibshirani 1994).

The remainder of the paper is organized as follows. Section 2 describes the motivating AML microbiome study and presents the proposed mediation framework and estimation procedures. Section 3 evaluates the performance of the proposed method through simulation studies and applies it to the AML microbiome data. Section 4 concludes with a discussion.

## 2 Materials and methods

### 2.1 The motivating study

Our work is motivated by an AML microbiome study conducted at MD Anderson, which aimed to understand the role of the microbiome in treatment-related infectious outcomes. The study enrolled 97 newly diagnosed adult patients with AML who received IC at MD Anderson between September 2013 and August 2015 (Galloway-Peña *et al.* 2020). Fecal specimens were collected at baseline (prior to IC initiation) and approximately every 96 hours during IC until neutrophil recovery, yielding 566 samples in total. Microbial DNA was extracted from stool samples, and the V4 region of the 16S rRNA gene was sequenced using the Illumina MiSeq platform. Sequencing reads were clustered into operational taxonomic units at a 97% similarity threshold and subsequently aggregated at the genus level to produce an abundance table for downstream analysis.

Our primary scientific question is whether gut microbiome features mediate the effect of IC intensity on infection status. As illustrated in Fig. 1, the exposure is a binary indicator of IC intensity, with high-intensity regimens (e.g. fludarabine-containing and other intensive therapies) coded as 1 and low-intensity regimens (e.g. hypomethylator-based combinations and low-dose cytarabine-based therapies) coded as 0 (Galloway-Peña *et al.* 2017). The outcome of interest is binary infection status during IC, defined using established microbiological or clinical criteria (Galloway-Peña *et al.* 2016, 2017). The mediator is the gut microbiome profile, represented by genus-level abundances measured from the stool sample collected immediately prior to infection onset or, for patients without infection, the last available sample. Mediation analysis in this study is complicated by antibiotic use during IC, which is commonly administered for infection prevention or treatment. Antibiotics are known to substantially alter gut microbiome composition (Schulfer *et al.* 2019) and may also directly influence infection risk (Schlesinger *et al.* 2009). We focus on broad-spectrum antibiotic use occurring between IC initiation and infection onset, which acts as an exposure-induced mediator–outcome confounder. In addition, we adjust for baseline covariates including age and sex.

**Figure 1:**
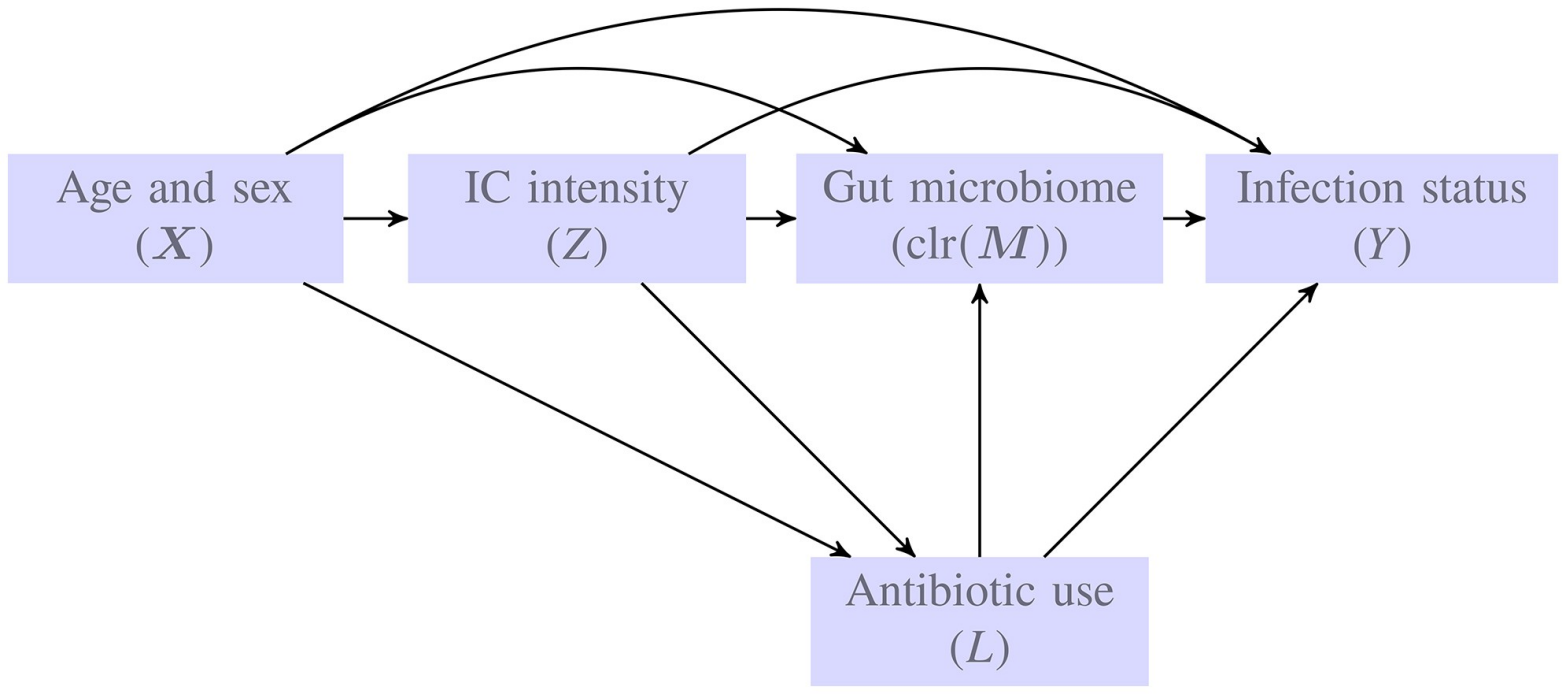
Conceptual causal diagram for the microbiome study of AML patients.

### 2.2 Methodology

#### 2.2.1 Preamble and notation

Let Z∈{0,1} denote a binary exposure, *Y* the outcome of interest, $M={\left(M_{1},\ldots ,M_{q}\right)}^{⊤}$ a vector of compositional mediators, *L* an exposure-induced mediator–outcome confounder, and X a vector of baseline covariates. We observe an independent and identically distributed (i.i.d.) sample of size *n*, ${\left\{(Z_{i},Y_{i},M_{i},L_{i},X_{i})\right\}}_{i=1}^{n}$.

Because $M_{i}$ is compositional, we apply the centered log-ratio (clr) transformation (Aitchison 1982, Lin and Peddada 2020), which maps the composition from the simplex to a (q−1)-dimensional Euclidean subspace: $\text{clr}(M_{i})=(\text{log}\;\{M_{i1}/g(M_{i})\},\ldots ,\;\text{log}\;\{M_{iq}/g(M_{i})\})$, where $g(M_{i})={\left(\prod_{k=1}^{q}M_{ik}\right)}^{1/q}$ denotes the geometric mean of the components of $M_{i}$. The clr-transformed components sum to zero, and each component represents the log-ratio of a taxon’s abundance to the geometric mean abundance across all taxa. By mapping the composition to a Euclidean subspace, the clr transformation facilitates the application of standard statistical methods to the transformed data (Lin and Peddada 2020). We use the clr transformation because it treats all components symmetrically without requiring the specification of a reference taxon and retains a direct correspondence between transformed features and genus-level taxa, thereby facilitating interpretation. In the subsequent analysis, we work with $\text{clr}(M_{i})$ in place of $M_{i}$.

Following the potential outcomes framework, let clr{M(z)} denote the clr-transformed mediator value that would have been observed if Z=z, and let Y{z,clr(m)} denote the outcome value that would have been observed if Z=z and clr(M)=clr(m). The potential outcome satisfies Y(z)=Y[z,clr{M(z)}]. The total effect of *Z* on *Y* is TE=E{Y(1)}−E{Y(0)}. Classical mediation analysis decomposes this effect into the natural direct effect (NDE) and natural indirect effect (NIE) (Robins and Greenland 1992, Pearl 2001):


$$\begin{array}{c} \text{NDE}=E\left[Y\left\{1,\text{clr}\left(M(0)\right)\right\}\right]-E\left[Y\left\{0,\text{clr}\left(M(0)\right)\right\}\right],\\ \text{NIE}=E\left[Y\left\{1,\text{clr}\left(M(1)\right)\right\}\right]-E\left[Y\left\{1,\text{clr}\left(M(0)\right)\right\}\right]. \end{array}$$

The NDE captures the component of the total effect not operating through the mediators, whereas the NIE represents the component mediated through clr(M). Under Assumptions S1–S5, available as supplementary data at *Bioinformatics* online, including the absence of exposure-induced mediator–outcome confounding (Assumption S5), these effects are identifiable via the mediation formula (Proposition S1, available as supplementary data at *Bioinformatics* online).

#### 2.2.2 Development under exposure-induced mediator–outcome confounding

As described in Section 2.1, the AML microbiome study involves an exposure-induced mediator–outcome confounder *L* (Fig. 1). Ignoring *L* leaves the mediator–outcome relationship confounded, whereas conditioning on *L* as a conventional confounder is also problematic because *L* is itself affected by the exposure. Consequently, Assumption S5, available as supplementary data at *Bioinformatics* online, required for identifying the natural effects is violated, and the NDE and NIE are generally non-identifiable. To address this setting, VanderWeele *et al.* (2014) introduced the interventional direct effect (IDE) and interventional indirect effect (IIE), defined as


$$\begin{array}{c} \text{IDE}=E\left[Y\left\{1,\mathrm{clr}(G(0|X))\right\}\right]-E\left[Y\left\{0,\mathrm{clr}(G(0|X))\right\}\right],\\ \text{IIE}=E\left[Y\left\{1,\mathrm{clr}(G(1|X))\right\}\right]-E\left[Y\left\{1,\mathrm{clr}(G(0|X))\right\}\right], \end{array}$$

where G(z∣X) denotes a random draw from the conditional distribution of M given X under exposure level *z*. Unlike the natural effects, the IDE and IIE remain identifiable without Assumption S5, available as supplementary data at *Bioinformatics* online; identification holds under Assumptions S1–S3 and S4a (Proposition S2), available as supplementary data at *Bioinformatics* online.

#### 2.2.3 Estimation of interventional direct and indirect effects

We develop an estimation procedure for the interventional direct and indirect effects that is tailored to the unique challenges of the AML microbiome study, including high-dimensional, zero-inflated, and dependent microbial mediators, as well as the presence of a binary exposure-induced mediator–outcome confounder (*L*, antibiotic use).

VanderWeele *et al.* (2014) suggested estimating the IIE using the formula


$$\begin{array}{c} \text{IIE}=E\left\{\frac{ZY}{\text{Pr}(Z=1|X)}\frac{\text{Pr}\{\text{clr}(M)|Z=1,X\}}{\text{Pr}\{\text{clr}(M)|Z=1,L,X\}}\right\}\\ -E\left\{\frac{ZY}{\text{Pr}(Z=1|X)}\frac{\text{Pr}\{\text{clr}(M)|Z=0,X\}}{\text{Pr}\{\text{clr}(M)|Z=1,L,X\}}\right\}. \end{array}$$

However, their approach requires modeling the conditional distributions Pr{clr(M)∣Z,X} and Pr{clr(M)∣Z,L,X}, which is infeasible in our AML microbiome study because the clr-transformed microbial mediators are high-dimensional and have complex dependence structure.

Instead, leveraging the identification results in Proposition S2, available as supplementary data at *Bioinformatics* online, we derive alternative identification formulas for the IDE and IIE that avoid modeling these mediator distributions. These formulas are given in Theorem 1 (Proof S3.3, available as supplementary data at *Bioinformatics* online).

Theorem 1. *Suppose that Supplementary Assumptions S1–S3, S4a, and S6 hold. Then IDE and IIE are identified as* 
 

$$\begin{array}{c} \text{IDE}=E\left[\frac{(1-Z)Y}{P(z_{0}|X)}\frac{E\{Y|z_{1},L,\text{clr}(M),X\}P(L|z_{1},X)}{E\{Y|z_{0},L,\text{clr}(M),X\}P(L|z_{0},\text{clr}(M),X)}\right]\\ -E\left[\frac{(1-Z)Y}{P(z_{0}|X)}\frac{P(L|z_{0},X)}{P\{L|z_{0},\text{clr}(M),X\}}\right], \end{array}$$

 

$$\begin{array}{c} \text{IIE}=E\left[\frac{ZY}{P(z_{1}|X)}\frac{P(L|z_{1},X)}{P\{L|z_{1},\text{clr}(M),X\}}\right]\\ -E\left[\frac{(1-Z)Y}{P(z_{0}|X)}\frac{E\{Y|z_{1},L,\text{clr}(M),X\}P(L|z_{1},X)}{E\{Y|z_{0},L,\text{clr}(M),X\}P\{L|z_{0},\text{clr}(M),X\}}\right]. \end{array}$$

where we use the shorthand $P(z_{i}|X)=\text{Pr}(Z=i|X)$, $E\{Y|z_{i},L,\text{clr}(M),X\}=E\{Y|Z=i,L,\text{clr}(M),X\}$, $P(L|z_{i},X)=\text{Pr}(L|Z=i,X)$ for i∈{0,1}, and $P\{L|z_{0},\text{clr}(M),X\}=\text{Pr}(L|Z=0,\text{clr}(M),X)$.

To further improve efficiency and interpretability, we restrict attention to the subset of clr-transformed mediators that are conditionally associated with the outcome given {Z,L,X}. This leads to simplified expressions for the IDE and IIE, formalized in Corollary 1 (Proof S3.4, available as supplementary data at *Bioinformatics* online).

Corollary 1. *Suppose that there exist subsets* $\text{clr}(M^{\left(1\right)})$  *and* $\text{clr}(M^{\left(2\right)})$ of the components of clr(M)* such that* $\text{clr}(M^{\left(1\right)})\cup \text{clr}(M^{\left(2\right)})=\text{clr}(M)$, $\text{clr}(M^{\left(1\right)})\cap \text{clr}(M^{\left(2\right)})=\emptyset$, $\text{clr}(M^{\left(1\right)})⫫\;/\;Y|\{Z,L,X\}$*, and*  $\text{clr}(M^{\left(2\right)})⫫Y|\{Z,L,X,\text{clr}(M^{\left(1\right)})\}$*. Both subsets retain the clr values computed from the full composition M. Assume that Supplementary Assumptions S1–S3, S4a, and S6a hold. Then IDE and IIE are identified as*
 

(1) $$\begin{array}{c} \text{IDE}=E[\frac{(1-Z)Y}{P(z_{0}|X)}\frac{E\{Y|z_{1},L,\text{clr}(M^{\left(1\right)}),X\}}{E\{Y|z_{0},L,\text{clr}(M^{\left(1\right)}),X\}}\\ \times \frac{P(L|z_{1},X)}{P\{L|z_{0},\text{clr}(M^{\left(1\right)}),X\}}]\\ -E[\frac{(1-Z)Y}{P(z_{0}|X)}\frac{P(L|z_{0},X)}{P\{L|z_{0},\text{clr}(M^{\left(1\right)}),X\}}]. \end{array}$$

 

(2) $$\begin{array}{c} \text{IIE}=E[\frac{ZY}{P(z_{1}|X)}\frac{P(L|z_{1},X)}{P\{L|z_{1},\text{clr}(M^{\left(1\right)}),X\}}]\\ -E[\frac{(1-Z)Y}{P(z_{0}|X)}\frac{E\{Y|z_{1},L,\text{clr}(M^{\left(1\right)}),X\}P(L|z_{1},X)}{E\{Y|z_{0},L,\text{clr}(M^{\left(1\right)}),X\}P\{L|z_{0},\text{clr}(M^{\left(1\right)}),X\}}]. \end{array}$$

*where we use the shorthand*  $E\{Y|z_{i},L,\text{clr}(M^{\left(1\right)}),X\}=E\{Y|Z=i,L,\text{clr}(M^{\left(1\right)}),X\}$  *for* i∈{0,1}, $P\{L|z_{0},\text{clr}(M^{\left(1\right)}),X\}=\text{Pr}(L|Z=0,\text{clr}(M^{\left(1\right)}),X)$*; all other shorthand notation follows Theorem 1*.

The IDE and IIE are then estimated based on (1) and (2). In the AML microbiome study, both *Z* and *L* are binary, so we model Pr(Z=1∣X) and Pr(L=1∣Z,X) using logistic regression with maximum likelihood estimation. Since *Y* is binary and the relevant mediator subset $\text{clr}(M^{\left(1\right)})$ is unknown a priori, we estimate Pr{Y=1∣Z,L,clr(M),X} using penalized logistic regression. The outcome model is constrained to include *Z*, *L*, and X, thereby ensuring that the estimation procedure is consistent with the identification formulas in (1) and (2). Let $\beta ={\left(\beta_{0},\beta_{Z},\beta_{L},\beta_{\text{clr}(M)}^{⊤},\beta_{X}^{⊤}\right)}^{⊤}$ denote the regression coefficients in the outcome model. For the tuning parameter λ, we define


$$\hat{\beta }(\lambda )=\underset{\beta }{\text{argmin}}\left[-\frac{2\;\text{log}\;\{L_{n}(\beta )\}}{n}+\sum_{k=1}^{q}p_{\lambda }(|\beta_{M_{k}}|)\right],$$

where $L_{n}(\beta )$ is the likelihood of the logistic regression model for *Y*, $\beta_{M_{k}}$ is the coefficient corresponding to the *k*th clr-transformed mediator, and $p_{\lambda }(\cdot )$ denotes the smoothly clipped absolute deviation (SCAD) penalty (Fan and Li 2001). The tuning parameter λ is selected by minimizing the Akaike Information Criterion (AIC) (Akaike 1974):


$$\hat{\lambda }=\text{arg}\underset{\lambda }{\min}\text{AIC}(\lambda )=\text{arg}\underset{\lambda }{\min}\left[-2\;\text{log}[L_{n}\{\hat{\beta }(\lambda )\}]+2\nu (\lambda )\right],$$

where ν(λ) is the number of nonzero coefficients in $\hat{\beta }(\lambda )$. The final estimator of β is chosen as $\hat{\beta }=\hat{\beta }(\hat{\lambda })$. The corresponding selected mediators are denoted by $\text{clr}({\hat{M}}^{\left(1\right)})$. Accordingly, the clr-transformed genus-level microbiome features selected by SCAD are regarded as candidate mediators within the joint mediation analysis.

Corollary 1 additionally requires estimation of $\text{Pr}\{L|Z,\text{clr}({\hat{M}}^{\left(1\right)}),X\}$. While we model Pr(L=1∣Z,X) using logistic regression, directly extending this model to include $\text{clr}({\hat{M}}^{\left(1\right)})$ may lead to model incompatibility. To avoid this issue, we estimate $\text{Pr}\{L|Z,\text{clr}({\hat{M}}^{\left(1\right)}),X\}$ using bagging with the optimal subset of deep neural networks (DNNs) (Mi *et al.* 2019), which flexibly captures nonlinear relationships and reduces overfitting. Implementation details are provided in Note S2.5, available as supplementary data at *Bioinformatics* online.

The final estimators of the IDE and IIE are obtained by plugging all component estimates into the identification formulas and computing empirical means. Algorithm S1, available as supplementary data at *Bioinformatics* online, summarizes the complete procedure for estimating the IIE; estimation of the IDE proceeds analogously. We note that the specific modeling choices here are motivated by the AML microbiome application, and alternative specifications may be preferable in other settings.

#### 2.2.4 Hypothesis testing

A key scientific question in the AML microbiome study is whether gut microbiome features mediate the effect of IC intensity on infection risk. This mediation effect can be quantified by the IIE defined in Section 2.2.2. Accordingly, we test $H_{0}:\text{IIE}=0\;\text{versus}\;H_{a}:\text{IIE}\neq 0$ at a prespecified significance level α. To conduct this test, we construct a 100(1−α)% bootstrap CI for the IIE using the standard normal bootstrap method (Efron and Tibshirani 1994). The null hypothesis is rejected if the resulting CI does not contain zero. This bootstrap-based testing procedure is straightforward to implement and can be efficiently accelerated using parallel computation when generating bootstrap replicates.

## 3 Results

### 3.1 Simulation studies

We conduct simulation studies to evaluate the finite-sample performance of the proposed method and examine two NIE-based reference approaches. The simulated data are generated according to the data-generating mechanism represented by the conceptual causal diagram in Fig. 1.

Baseline covariates $X={\left(X_{1},X_{2}\right)}^{⊤}$, representing age and sex (1 = male; 0 = female), are sampled with replacement from the AML microbiome dataset; age is divided by 100 to place it on a scale comparable to sex. Conditional on X, the binary exposure *Z* and the exposure-induced mediator–outcome confounder *L* are generated from logistic regression models $\text{Pr}(Z=1|X)=\text{expit}(\alpha_{0}+\alpha_{X}^{⊤}X)$ and $\text{Pr}(L=1|Z,X)=\text{expit}(\gamma_{0}+\gamma_{Z}Z+\gamma_{X}^{⊤}X)$, respectively, where expit(x)=exp(x)/{1+exp(x)}. To facilitate computation of the true IIE, we generate the clr-transformed mediators directly. Let $\text{clr}(M)≜{\left({\text{clrM}}_{1},\ldots ,{\text{clrM}}_{q}\right)}^{⊤}$. We first generate $C|Z,L,X∼\text{Gamma}\{\eta_{0},\theta (Z,L,X)\}$, where $\eta_{0}$ is the shape parameter and $\theta (Z,L,X)=\text{exp}(\theta_{0}+\theta_{Z}Z+\theta_{L}L+\theta_{X}^{⊤}X)/\eta_{0}$ is the scale parameter. We then generate ζ∼DiscreteUniform{⌊(q−1)a⌋,…,⌊(q−1)b⌋}, where ⌊·⌋ denotes the floor function. Conditional on *C* and ζ, ζ of the first q−1 clr-transformed mediators are randomly selected and assigned the value −C, corresponding to taxa with zero read counts before zero replacement and clr transformation. The remaining q−1−ζ components are generated independently from


$$\text{Uniform}\left(\frac{-C(1-\zeta )}{q-1-\zeta },\frac{(1+\zeta )C}{q-1-\zeta }\right).$$

The final component is set to ${\text{clrM}}_{q}=-\sum_{k=1}^{q-1}{\text{clrM}}_{k}$ to enforce the inherent sum-to-zero constraint. This construction is chosen to approximately reproduce the zero-inflation pattern prior to clr transformation and other key characteristics of the clr-transformed microbiome data observed in the AML study. A detailed explanation of the rationale underlying the proposed data-generation mechanism is provided in Note S2.6, available as supplementary data at *Bioinformatics* online. The binary outcome *Y* is generated from a logistic regression model $\text{Pr}(Y=1|Z,L,\text{clr}(M),X)=\text{expit}(\beta_{0}+\beta_{Z}Z+\beta_{L}L+\beta_{\text{clr}(M)}^{⊤}\text{clr}(M)+\beta_{X}^{⊤}X).$

We set the model parameters as follows: $\alpha_{0}=0.2$, $\alpha_{X}={\left(-0.5,0.5\right)}^{⊤}$, $\gamma_{0}=0.5$, $\gamma_{Z}=1$, $\gamma_{X}={\left(0.5,-0.5\right)}^{⊤}$, $\theta_{0}=-1$, $\theta_{Z}=2$, $\theta_{L}=-0.2$, $\theta_{X}={\left(-0.8,-0.2\right)}^{⊤}$, $\eta_{0}=4$, a=0.4, b=0.8, $\beta_{0}=3$, $\beta_{Z}=-1.2$, $\beta_{L}=-6$, $\beta_{X}={\left(-1.2,-1.2\right)}^{⊤}$, $\beta_{\text{clr}(M)}={\left(-8,-8,0,\ldots ,0\right)}^{⊤}$ with q=134, and n=70. Together, these parameter values define the baseline simulation setting. The primary sample size (n=70) and the number of clr-transformed mediators (q=134) are chosen to match those of the AML microbiome study. The remaining model parameters are selected with reference to summary measures observed in the AML microbiome data. Table S1, available as supplementary data at *Bioinformatics* online reports these observed summary measures alongside their corresponding means across 500 datasets generated under the baseline simulation setting, providing a descriptive comparison between the observed and simulated data.

To investigate performance across a range of scientifically plausible scenarios, we consider additional simulation settings by varying the sample size and the parameters governing the effects along the paths Z→L, Z→clr(M), and L→clr(M). Specifically, we let $\gamma_{Z}\in \{0,1\}$, representing the absence or presence of an effect along the path Z→L. The parameters $\theta_{Z}\in \{0,1.5,2\}$ and $\theta_{L}\in \{0,-0.1,-0.2\}$ control the effects along the paths Z→clr(M) and L→clr(M), respectively. In particular, $\theta_{Z}$ controls whether *Z* directly affects the distribution of the clr-transformed mediators through *C*, conditional on *L* and X. It does not parameterize the number of taxa whose underlying absolute abundances are directly affected by the exposure; further discussion of this distinction is provided in Note S2.6, available as supplementary data at *Bioinformatics* online. The coefficient vector $\beta_{\text{clr}(M)}$ is set to either ${\left(0,\ldots ,0\right)}^{⊤}$ or ${\left(-8,-8,0,\ldots ,0\right)}^{⊤}$, corresponding to scenarios in which either none or only a subset of clr-transformed mediators affect *Y*. An additional sample size of n=200 is also considered to assess the impact of increasing sample size.

In addition to the proposed method, we consider two NIE-based reference approaches. The first approach is constructed by adapting the proposed estimation procedure to target the NIE while ignoring the exposure-induced mediator–outcome confounder *L*. Details are provided in Note S2.7, available as supplementary data at *Bioinformatics* online, and we refer to this approach as the IPW-NIE method. The second approach is the existing compositional mediation method of Sohn *et al.* (2022), implemented using the R package cmmb. Although this method also targets the NIE, its estimation strategy differs substantially from that of the IPW-NIE method. We refer to this approach as the Sohn-NIE method.

Results are summarized over 500 Monte Carlo replications, with 400 bootstrap replications used for each method in each simulated dataset. Performance is assessed using bias, standard deviation (SD), root mean squared error (RMSE), and the type I error rate or empirical power at the significance level α=0.05. For all methods, bias and RMSE are calculated relative to the true IIE. In settings where the NIE and IIE do not coincide, these quantities for the NIE-based reference approaches represent descriptive discrepancies from the IIE rather than bias and RMSE for estimation of their NIE estimand. The proposed and IPW-NIE methods use standard normal bootstrap CIs, whereas the Sohn-NIE method uses the empirical bootstrap CI implemented by ci.method = “empirical” in the cmmb package. Alternative bootstrap CIs for the proposed and IPW-NIE methods are compared in Note S2.8 and Tables S2–S4, available as supplementary data at *Bioinformatics* online.

Table 1 reports results under scenarios where $\gamma_{Z}=\theta_{Z}=0$ and/or $\beta_{\text{clr}(M)}={\left(0,\ldots ,0\right)}^{⊤}$. The IIE is identifiable and equals zero in all scenarios considered. When $\gamma_{Z}=\theta_{Z}=0$, the NIE is also identifiable and equals zero. In the scenarios with $\theta_{Z}\neq 0$ considered here, the assumptions required for identification of the NIE are violated, whereas the IIE remains identifiable and equals zero. When $\gamma_{Z}=\theta_{Z}=0$, both the proposed method and the IPW-NIE method maintain type I error rates close to the nominal level of 0.05 for both sample sizes, while the proposed method consistently achieves smaller RMSE values. In contrast, the Sohn-NIE method exhibits inflated type I error rates when n=70, although its performance improves as the sample size increases. Across all methods, the absolute biases are generally small relative to their corresponding SDs. When $\theta_{Z}\neq 0$, the proposed method continues to maintain type I error rates close to the nominal level for both sample sizes. However, the type I error rates of the IPW-NIE and Sohn-NIE methods deviate substantially from the nominal level. Moreover, in these scenarios where the NIE is not identifiable, the proposed method consistently exhibits substantially smaller absolute bias than both NIE-based reference approaches.

**Table 1:** Bias, SD, RMSE, and type I error rate for testing $H_{0}:\text{mediation}\;\text{effect}=0$ versus $H_{a}:\text{mediation}\;\text{effect}\neq 0$ at the significance level α=0.05 under zero-mediation-effect scenarios.^a^

*n*	$\theta_{L}$	$\gamma_{Z}$	$\theta_{Z}$	$\beta_{\text{clr}(M)}^{⊤}$	Proposed method				IPW-NIE method				Sohn-NIE method				
					Bias	SD	RMSE	Type I error rate	Bias	SD	RMSE	Type I error rate	Bias	SD	RMSE	Type I error rate	$N_{\text{NA}}^{\;}$ ^b^
70	0	0	0	(0,…,0)	0.77	7.05	7.09	0.024	0.42	9.27	9.27	0.024	−3.19	8.03	8.63	0.126	0
70	−0.1	0	0	(0,…,0)	0.85	6.89	6.94	0.022	0.27	9.26	9.25	0.022	−3.36	8.19	8.84	0.122	0
70	−0.2	0	0	(0,…,0)	1.02	6.84	6.91	0.020	0.78	9.56	9.58	0.026	−3.56	8.43	9.14	0.142	0
70	0	0	0	(−8,−8,0,…,0)	1.68	8.96	9.11	0.020	0.74	10.16	10.18	0.026	−1.94	8.62	8.82	0.102	0
70	−0.1	0	0	(−8,−8,0,…,0)	1.67	8.45	8.60	0.024	1.23	10.38	10.45	0.036	−1.80	8.73	8.91	0.100	0
70	−0.2	0	0	(−8,−8,0,…,0)	1.41	8.37	8.48	0.026	1.00	9.78	9.82	0.034	−1.94	8.32	8.54	0.088	0
70	−0.2	0	1.5	(0,…,0)	1.68	7.57	7.74	0.028	13.36	8.99	16.10	0.248	−11.77	6.79	13.59	0.622	0
70	−0.2	0	2	(0,…,0)	0.98	8.09	8.14	0.030	12.66	12.63	17.87	0.184	−17.93	6.11	18.94	0.883	3
70	−0.2	1	1.5	(0,…,0)	−2.80	3.85	4.76	0.026	7.67	5.59	9.49	0.110	−16.57	5.40	17.43	0.928	0
70	−0.2	1	2	(0,…,0)	−2.29	4.40	4.95	0.038	8.65	5.69	10.35	0.208	−18.03	5.18	18.76	0.978	1
200	0	0	0	(0,…,0)	0.58	4.60	4.63	0.046	1.81	8.64	8.82	0.032	−1.86	3.58	4.03	0.056	0
200	−0.1	0	0	(0,…,0)	0.67	4.48	4.53	0.030	1.53	9.85	9.96	0.040	−1.95	3.68	4.16	0.056	0
200	−0.2	0	0	(0,…,0)	0.40	4.43	4.45	0.034	1.47	8.76	8.87	0.028	−2.23	3.74	4.35	0.068	0
200	0	0	0	(−8,−8,0,…,0)	1.00	5.87	5.95	0.060	0.49	10.42	10.42	0.044	−1.22	3.97	4.15	0.034	0
200	−0.1	0	0	(−8,−8,0,…,0)	0.85	5.34	5.41	0.038	0.59	10.51	10.52	0.030	−0.98	4.06	4.17	0.038	0
200	−0.2	0	0	(−8,−8,0,…,0)	0.85	5.36	5.42	0.046	0.93	8.87	8.91	0.030	−1.07	4.05	4.18	0.040	0
200	−0.2	0	1.5	(0,…,0)	1.33	6.01	6.14	0.060	17.50	5.66	18.39	0.806	−4.73	3.26	5.74	0.416	0
200	−0.2	0	2	(0,…,0)	1.43	6.25	6.41	0.060	15.19	8.74	17.52	0.626	−9.55	2.94	9.99	0.942	2
200	−0.2	1	1.5	(0,…,0)	−1.47	3.48	3.78	0.086	8.93	3.63	9.64	0.832	−6.61	2.13	6.94	0.956	0
200	−0.2	1	2	(0,…,0)	−1.22	3.85	4.03	0.074	10.03	3.65	10.68	0.922	−7.08	2.04	7.37	0.976	0

a All reported bias, SD, and RMSE values are multiplied by 100. For all methods, bias and RMSE are calculated relative to zero.

b $N_{\text{NA}}$ denotes the number of Monte Carlo replications (out of 500) for which the Sohn-NIE method failed because of computational issues (e.g. non-invertible matrices), resulting in missing (NA) estimates. Bias, SD, RMSE, and type I error rate for this method are computed after excluding these failed replications.

Table 2 summarizes the results under scenarios with a nonzero mediation effect. In these scenarios, the assumptions required for identification of the NIE are violated, whereas the IIE remains identifiable. Overall, the proposed method consistently exhibits smaller absolute bias and RMSE than both NIE-based reference approaches. The power of the proposed method increases with both $\gamma_{Z}$ and sample size. Similar trends are observed for the IPW-NIE method. However, its power is generally lower than that of the proposed method when n=200. In contrast, the Sohn-NIE method exhibits substantially larger absolute bias and RMSE and maintains very low power across all scenarios, even when n=200. Moreover, the Sohn-NIE method may encounter computational failures, resulting in missing estimates in some Monte Carlo replications. The frequency of such failures increases markedly when $\theta_{Z}=2$ and is particularly severe when n=200.

**Table 2:** The true value of the IIE, denoted by Truth, bias, SD, RMSE, and empirical power for testing $H_{0}:\text{mediation}\;\text{effect}=0$ versus $H_{a}:\text{mediation}\;\text{effect}\neq 0$ at the significance level α=0.05 under nonzero-mediation-effect scenarios with $\theta_{L}=-0.2$ and $\beta_{\text{clr}(M)}^{⊤}=(-8,-8,0,\ldots ,0)$.^a^

*n*	$\gamma_{Z}$	$\theta_{Z}$	Proposed method					IPW-NIE method				Sohn-NIE method				
			Truth	Bias	SD	RMSE	Power	Bias	SD	RMSE	Power	Bias	SD	RMSE	Power	$N_{\text{NA}}^{\;}$ ^b^
70	0	1.5	15.09	0.35	11.02	11.01	0.224	3.11	13.62	13.96	0.302	−17.09	8.86	19.25	0.150	1
70	0	2	17.20	−0.41	11.63	11.63	0.244	2.50	13.63	13.85	0.250	−20.41	8.63	22.15	0.171	14
70	1	1.5	20.76	−0.53	11.01	11.01	0.416	−0.57	11.20	11.21	0.416	−23.68	8.59	25.19	0.194	0
70	1	2	23.95	−1.56	11.26	11.36	0.424	−1.83	11.58	11.71	0.368	−28.07	8.58	29.35	0.189	13
200	0	1.5	15.09	1.22	7.09	7.19	0.630	6.24	10.51	12.22	0.558	−14.62	4.11	15.18	0.037	10
200	0	2	17.20	0.85	7.59	7.63	0.652	5.53	9.81	11.26	0.450	−17.76	4.31	18.27	0.055	81
200	1	1.5	20.76	−0.08	6.11	6.10	0.884	2.78	6.91	7.44	0.836	−20.57	4.01	20.95	0.047	8
200	1	2	23.95	−1.25	6.63	6.74	0.884	1.31	7.30	7.41	0.776	−24.78	4.11	25.12	0.058	71

a All reported truth, bias, SD, and RMSE values are multiplied by 100. For all methods, bias and RMSE are calculated relative to the true IIE.

b $N_{\text{NA}}$ denotes the number of Monte Carlo replications (out of 500) for which the Sohn-NIE method failed because of computational issues (e.g. non-invertible matrices), resulting in missing (NA) estimates. Bias, SD, RMSE, and power for this method are computed after excluding these failed replications.

### 3.2 Analysis of the AML microbiome data

We apply the proposed method to the AML microbiome data to assess whether gut microbiome features mediate the causal effect of IC intensity on infection status, adjusting for the exposure-induced mediator–outcome confounder, antibiotic use, and baseline covariates age and sex (Fig. 1; Section 2.1). We include 70 patients with at least one stool sample collected between IC initiation and either infection onset or last follow-up. For each patient, we use the sample collected immediately prior to infection onset or, for patients without infection, the last available sample. The mean age was 56.2 years (SD: 15.2), and 33 patients were male.

Following standard microbiome filtering procedures (Lu *et al.* 2019), taxa with low abundance are excluded. Specifically, we retain genus-level taxa present in at least 10% of the samples, resulting in 134 genus-level features for analysis. Zero counts are replaced by the maximum rounding error of 0.5, a common practice in the analysis of compositional and microbiome data (Shi *et al.* 2016). The read counts are then converted to genus-level compositions and transformed using the clr transformation.

Among the 70 patients, 46 patients received high-intensity IC and 24 patients received low-intensity IC. In the high-intensity group, 39 patients used broad-spectrum antibiotics and 15 developed infections; in the low-intensity group, 14 patients used broad-spectrum antibiotics and 8 developed infections. We estimate the average treatment effect of IC intensity on infection status using the Horvitz-Thompson estimator (Horvitz and Thompson 1952), with the propensity score Pr(Z=1|X) modeled via logistic regression. After adjusting for age and sex, the high-intensity IC is associated with a 23.5-percentage-point higher infection risk (95% standard normal bootstrap CI: [−8.1,55.1] percentage points). All bootstrap CIs in the real-data analysis were constructed using 400 bootstrap replications.

We then apply the proposed mediation framework (Sections 2.2.3 and 2.2.4) to investigate whether the effect of IC intensity on infection status is mediated through gut microbiome features. For comparison, we also implement the IPW-NIE method and the Sohn-NIE method described in Section 3.1, along with their corresponding approaches for estimating the NDE (the IPW-NDE method and the Sohn-NDE method). Table 3 presents the estimated effects and 95% bootstrap CIs. The proposed method indicates a significant IIE through changes in the gut microbiome at α=0.05, whereas the estimated IDE is smaller in magnitude and is not significantly different from zero. In contrast, neither the NIE nor the NDE is significantly different from zero when using methods that ignore the exposure-induced mediator–outcome confounder, underscoring the importance of accounting for antibiotic use. The computational costs of different methods are provided in Note S2.9, available as supplementary data at *Bioinformatics* online.

**Table 3:** Estimated mediation and direct effects with 95% bootstrap CIs, adjusted for baseline covariates X.

Effect	Estimate	95% CI
Proposed method for IIE	0.403	[0.068, 0.738]
Proposed method for IDE	−0.063	[−0.205, 0.079]
IPW-NIE method	0.252	[−0.145, 0.649]
IPW-NDE method	−0.017	[−0.352, 0.318]
Sohn-NIE method	0.021	[−0.248, 0.258]
Sohn-NDE method	0.022	[−0.017, 0.065]

The penalized logistic regression outcome model selected 11 clr-transformed microbial features from the genus-level abundance table as candidate mediators (Table S5, available as supplementary data at *Bioinformatics* online). Several selected features map to taxonomic groups containing members previously reported to change during chemotherapy-based cancer treatment or to be associated with infection or infection-related outcomes; the relevant literature is summarized in Note S2.10, available as supplementary data at *Bioinformatics* online. These reported associations provide biological context but should not be interpreted as evidence of taxon-specific causal mediation effects.

To examine sensitivity to baseline covariate adjustment, which is relevant to the assumptions of no unmeasured confounding required for identification under the proposed method (Assumptions S2, S3, and S4a, available as supplementary data at *Bioinformatics* online), we repeat the analysis without adjusting for the baseline covariates X (Table S6, available as supplementary data at *Bioinformatics* online). With adjustment for X, the IIE is significantly different from zero and the IDE estimate is negative. Without adjustment, the IIE is no longer significant and the IDE estimate becomes positive. These differences indicate that adjustment for the measured baseline covariates materially affects the estimated effects and inferential conclusions, underscoring the importance of adequate confounding control.

We also assess the sensitivity of the results to the zero-count replacement value and the choice of log-ratio transformation. The conclusion of a significant IIE and a nonsignificant IDE remains unchanged across the zero-count replacement values of 0.1, 0.5, and 0.9 and across the clr, additive log-ratio (alr), and isometric log-ratio (ilr) transformations. Detailed results are provided in Note S2.11 and Tables S7 and S8, available as supplementary data at *Bioinformatics* online.

## 4 Discussion

In this paper, we investigate the causal relationships among IC intensity, infection status, and the on-treatment gut microbiome using data from an AML microbiome study conducted at MD Anderson. To account for antibiotic use, which is induced by IC intensity and may confound the microbiome–infection relationship, we adopt the interventional indirect effect framework. To accommodate the high dimensionality, zero inflation, and dependence of microbial mediators, we develop novel identification formulas and corresponding estimation procedures for the interventional effects. Our approach combines sparsity-inducing regularization with the clr transformation to handle compositional microbial data. Mediation effects are assessed using standard normal bootstrap CIs. Simulation studies demonstrate that the proposed method yields accurate effect estimates and maintains appropriate type I error rates and power. Application to the AML microbiome data provides evidence of a statistically significant IIE of IC intensity on infection status through changes in the gut microbiome, with 11 clr-transformed microbial features selected as candidate mediators.

In the present study, we focus on microbiome measurements from a single clinically relevant time point. However, the AML microbiome study collected repeated stool samples throughout the IC treatment period. Considering all the measurements in the analysis could provide additional insight but would substantially increase the complexity of mediation analysis (Gilbert *et al.* 2025). We plan to investigate this extension in our future research. In addition, our analysis targets the joint mediation effect through the selected microbiome features. In many applications, it is also of interest to quantify the mediation effect of each individual mediator. This is challenging because mediators may be conditionally dependent or causally ordered, in which case some mediators can themselves act as exposure-induced mediator–outcome confounders for others (Zhou 2022). Addressing these issues is nontrivial and will be explored in future work.

## Supplementary Material

btag750_Supplementary_Data

## Data Availability

The gut microbiome sequencing data supporting the findings of this article are available in the NCBI Sequence Read Archive under BioProject ID PRJNA352060 (http://www.ncbi.nlm.nih.gov/sra). Clinical metadata for the AML microbiome cohort are available through the Supplementary Materials of Galloway-Peña *et al.* (2020) at https://pmc.ncbi.nlm.nih.gov/articles/instance/7312220/bin/ciz777_suppl_supplementary_material.pdf. The R code used for the simulation studies is available at https://github.com/YX-ZHANG-1/CausalMediationMicrobiome, and the version used to produce the results reported in this manuscript (v1.0.0) is archived on Zenodo at https://doi.org/10.5281/zenodo.21960802.
