# Supplementary material for: Inverse probability weighting-based mediation analysis for microbiome data
Source: Bioinformatics. 2026 Oct 13;42(10):btag750. doi: 10.1093/bioinformatics/btag750 (PMC13714993; doi:10.1093/bioinformatics/btag750)
Supplement: Supplementary file 1 [file btag750_supplementary_data.zip › Files_BKup/21-Oct-2026_040439_Supplementary_materials.pdf]

Supplementary materials for
"Inverse probability weighting-based mediation analysis for microbiome data"

Yuexia Zhang[1], Jian Wang[2], Jiayi Shen[3], Jessica Galloway-Peña[4], Samuel Shelburne[5], Linbo Wang[6*], and Jianhua Hu[7*]

[1]Department of Statistics and Data Science, The University of Texas at San Antonio, San Antonio, TX 78207, United States
[2]Department of Biostatistics, The University of Texas MD Anderson Cancer Center, Houston, TX 77030, United States
[3]Statistics for Clinical Development – US, BeOne Medicines, San Carlos, CA 94070, United States
[4]Department of Veterinary Pathobiology, Texas A&M University, College Station, TX 77843, United States
[5]Department of Infectious Diseases, Infection Control and Employee Health, The University of Texas MD Anderson Cancer Center, Houston, TX 77030, United States
[6]Department of Statistical Sciences, University of Toronto, Toronto, ON M5G 1Z5, Canada
[7]Department of Biostatistics, Columbia University, New York, NY 10032, United States
[*]Corresponding authors: Linbo Wang, E-mail: linbo.wang@utoronto.ca; Jianhua Hu, E-mail: jh3992@cumc.columbia.edu.

S1. Supplementary Tables

Supplementary Table S1. Comparison of selected characteristics between the AML microbiome study and the baseline simulation setting with $\gamma_Z = 1$, $\theta_Z = 2$, $\theta_L = -0.2$, $\boldsymbol{\beta}_{\mathrm{clr}(\boldsymbol{M})} = (-8, -8, 0, \ldots, 0)^\top$, and $n = 70$.

Characteristic	AML study	Simulation mean^a
Baseline covariates		
Mean of X_1	0.562	0.562
SD of X_1	0.152	0.151
$\Pr(X_2 = 1)$	0.471	0.472
Exposure, exposure-induced mediator–outcome confounder, and outcome		
$\Pr(Z = 1)$	0.657	0.538
$\Pr(L = 1)$	0.757	0.737
$\Pr(Y = 1)$	0.329	0.357
Microbiome characteristics		
Mean of C	0.959	0.815
Mean proportion of zero counts	0.720	0.598
25th percentile of pairwise correlations for $\mathrm{clr}(\boldsymbol{M})$	-0.131	-0.190
Median of pairwise correlations for $\mathrm{clr}(\boldsymbol{M})$	-0.013	-0.023
75th percentile of pairwise correlations for $\mathrm{clr}(\boldsymbol{M})$	0.119	0.163

^{a} Simulation means are calculated across 500 simulated datasets generated under the baseline simulation setting.

Supplementary Table S2. Coverage probabilities of 95% bootstrap confidence intervals (CIs) for the mediation effect under zero-mediation-effect scenarios.[a]

					Proposed method			IPW-NIE method		
n	θ_L	γ_Z	θ_Z	$\boldsymbol{\beta}^{\top}_{\mathrm{clr}(\boldsymbol{M})}$	Percentile CI	BCa CI	Normal CI	Percentile CI	BCa CI	Normal CI
70	0	0	0	$(0,\ldots,0)$	0.992	0.986	0.976	0.998	0.996	0.976
70	-0.1	0	0	$(0,\ldots,0)$	0.990	0.984	0.978	1.000	0.998	0.978
70	-0.2	0	0	$(0,\ldots,0)$	0.990	0.986	0.980	0.998	0.994	0.974
70	0	0	0	$(-8,-8,0,\ldots,0)$	0.988	0.986	0.980	0.998	0.998	0.974
70	-0.1	0	0	$(-8,-8,0,\ldots,0)$	0.992	0.984	0.976	0.996	0.998	0.964
70	-0.2	0	0	$(-8,-8,0,\ldots,0)$	0.996	0.990	0.974	0.998	0.998	0.966
70	-0.2	0	1.5	$(0,\ldots,0)$	0.996	0.986	0.972	0.914	0.906	0.752
70	-0.2	0	2	$(0,\ldots,0)$	0.994	0.980	0.970	0.950	0.940	0.816
70	-0.2	1	1.5	$(0,\ldots,0)$	0.952	0.934	0.974	0.910	0.866	0.890
70	-0.2	1	2	$(0,\ldots,0)$	0.962	0.940	0.962	0.846	0.778	0.792
200	0	0	0	$(0,\ldots,0)$	0.998	0.998	0.954	1.000	1.000	0.968
200	-0.1	0	0	$(0,\ldots,0)$	0.998	0.998	0.970	0.996	0.998	0.960
200	-0.2	0	0	$(0,\ldots,0)$	0.998	0.998	0.966	1.000	1.000	0.972
200	0	0	0	$(-8,-8,0,\ldots,0)$	0.988	0.986	0.940	1.000	1.000	0.956
200	-0.1	0	0	$(-8,-8,0,\ldots,0)$	0.988	0.988	0.962	1.000	1.000	0.970
200	-0.2	0	0	$(-8,-8,0,\ldots,0)$	0.996	0.994	0.954	1.000	1.000	0.970
200	-0.2	0	1.5	$(0,\ldots,0)$	0.998	1.000	0.940	0.248	0.364	0.194
200	-0.2	0	2	$(0,\ldots,0)$	0.994	0.994	0.940	0.390	0.508	0.374
200	-0.2	1	1.5	$(0,\ldots,0)$	0.972	0.970	0.914	0.152	0.180	0.168
200	-0.2	1	2	$(0,\ldots,0)$	0.970	0.964	0.926	0.036	0.042	0.078

[a] Percentile CI denotes the bootstrap percentile CI, BCa CI denotes the bias-corrected and accelerated bootstrap CI, and Normal CI denotes the standard normal bootstrap CI.

Supplementary Table S3. The true value of the interventional indirect effect (IIE), denoted by Truth, and coverage probabilities of 95% bootstrap CIs for the mediation effect under nonzero-mediation-effect scenarios with $\theta_L = -0.2$ and $\boldsymbol{\beta}^{\top}_{\mathrm{clr}(\boldsymbol{M})} = (-8,-8,0,\ldots,0)$.[a]

			Proposed method				IPW-NIE method		
n	γ_Z	θ_Z	Truth	Percentile CI	BCa CI	Normal CI	Percentile CI	BCa CI	Normal CI
70	0	1.5	15.09	0.990	0.988	0.958	1.000	1.000	0.966
70	0	2	17.20	0.990	0.984	0.964	1.000	1.000	0.976
70	1	1.5	20.76	0.964	0.954	0.950	0.996	0.994	0.984
70	1	2	23.95	0.970	0.968	0.954	0.998	0.992	0.974
200	0	1.5	15.09	0.986	0.986	0.960	0.952	0.966	0.892
200	0	2	17.20	0.986	0.988	0.944	0.990	0.992	0.954
200	1	1.5	20.76	0.988	0.986	0.968	0.984	0.988	0.964
200	1	2	23.95	0.978	0.986	0.958	1.000	0.998	0.986

[a] Percentile CI denotes the bootstrap percentile CI, BCa CI denotes the bias-corrected and accelerated bootstrap CI, and Normal CI denotes the standard normal bootstrap CI. All reported truth values are multiplied by 100.

Supplementary Table S4. The true value of the interventional indirect effect (IIE), denoted by Truth, and empirical power for testing H_0 : mediation effect $= 0$ versus H_a : mediation effect $\neq 0$ at the significance level $\alpha = 0.05$ under nonzero-mediation-effect scenarios with $\theta_L = -0.2$ and $\beta_{\mathrm{clr}(M)}^{\top} = (-8, -8, 0, \ldots, 0)$.[a]

			Proposed method				IPW-NIE method		
n	γ_Z	θ_Z	Truth	Percentile CI	BCa CI	Normal CI	Percentile CI	BCa CI	Normal CI
70	0	1.5	15.09	0.126	0.120	0.224	0.118	0.102	0.302
70	0	2	17.20	0.124	0.142	0.244	0.128	0.140	0.250
70	1	1.5	20.76	0.322	0.344	0.416	0.204	0.176	0.416
70	1	2	23.95	0.358	0.360	0.424	0.200	0.188	0.368
200	0	1.5	15.09	0.564	0.578	0.630	0.586	0.510	0.558
200	0	2	17.20	0.664	0.686	0.652	0.512	0.448	0.450
200	1	1.5	20.76	0.904	0.896	0.884	0.874	0.826	0.836
200	1	2	23.95	0.940	0.944	0.884	0.850	0.796	0.776

[a] Percentile CI denotes the bootstrap percentile CI, BCa CI denotes the bias-corrected and accelerated bootstrap CI, and Normal CI denotes the standard normal bootstrap CI. All reported truth values are multiplied by 100.

Supplementary Table S5. Details of the selected microbial features in the AML microbiome data analysis.[a]

OTU	Genus	Family	Kingdom
GFQLact9	*Lactococcus*	Streptococcaceae	Bacteria
GG7The26	*Bacteroides*	Bacteroidaceae	Bacteria
GWTAmn51	*Enterobacter*	Enterobacteriaceae	Bacteria
Unc03xpn	*Lachnoclostridium*	Lachnospiraceae	Bacteria
Unc04n2w	Actinomycetaceae (Unc04n2w)	Actinomycetaceae	Bacteria
Unc05m27	Ruminococcaceae (Unc05m27)	Ruminococcaceae	Bacteria
Unc05mrd	*Akkermansia*	Verrucomicrobiaceae	Bacteria
Unc36622	Lachnospiraceae (Unc36622)	Lachnospiraceae	Bacteria
Unc94755	*Alistipes*	Rikenellaceae	Bacteria
UncCl291	Clostridiales (UncCl291)	Clostridiales (UncCl291)	Bacteria
UncM5880	*Methanobrevibacter*	Methanobacteriaceae	Archaea

[a] Taxonomic annotations are reproduced from the original taxon table. For features not classified at the genus level, the available higher-level taxonomic annotation is followed by the original feature identifier in parentheses. "Clostridiales (UncCl291)" is retained as the family-level annotation assigned in the original data.

Supplementary Table S6. Estimated interventional indirect and direct effects (IIE and IDE) with 95% bootstrap CIs, unadjusted for baseline covariates $\boldsymbol{X}$.

Effect	Estimate	95% CI
Proposed method for IIE	0.067	[-0.183, 0.318]
Proposed method for IDE	0.023	[-0.251, 0.296]

Supplementary Table S7. Estimated interventional indirect and direct effects (IIE and IDE) with 95% bootstrap CIs under different zero-count replacement values, adjusted for baseline covariates $\boldsymbol{X}$.

Zero-count replacement value	Effect	Estimate	95% CI
0.1	Proposed method for IIE	0.443	[0.102, 0.784]
	Proposed method for IDE	-0.115	[-0.262, 0.031]
0.5	Proposed method for IIE	0.403	[0.068, 0.738]
	Proposed method for IDE	-0.063	[-0.205, 0.079]
0.9	Proposed method for IIE	0.442	[0.111, 0.773]
	Proposed method for IDE	-0.109	[-0.249, 0.031]

Supplementary Table S8. Estimated interventional indirect and direct effects (IIE and IDE) with 95% bootstrap CIs under different log-ratio transformations of $\boldsymbol{M}$, adjusted for baseline covariates $\boldsymbol{X}$.[a]

Transformation of $\boldsymbol{M}$	Effect	Estimate	95% CI
clr	IIE	0.403	[0.068, 0.738]
	IDE	-0.063	[-0.205, 0.079]
alr	IIE	0.403	[0.049, 0.756]
	IDE	-0.023	[-0.170, 0.125]
ilr	IIE	0.400	[0.061, 0.740]
	IDE	-0.067	[-0.211, 0.077]

[a] The clr transformation corresponds to the transformation used in the proposed method. For the additive log-ratio (alr) transformation, the last genus-level taxon, unide146, is used as the reference component. For the isometric log-ratio (ilr) transformation, the clr-transformed composition is projected onto the basic (Helmert) orthonormal basis implemented by `ilrBase()` with `method = "basic"` in the R package `compositions`, yielding $q-1$ ilr coordinates.

S2. Supplementary Notes

S2.1. Regularity conditions

Assumption S1 (Consistency) $\text{clr}(\boldsymbol{M}) = \text{clr}\{\boldsymbol{M}(z)\}$ when $Z = z$; $Y = Y\{z, \text{clr}(\boldsymbol{m})\}$ when $Z = z$ and $\text{clr}(\boldsymbol{M}) = \text{clr}(\boldsymbol{m})$.

Assumption S2 (No unmeasured $Z - Y$ confounding) For all $z, \boldsymbol{m}$, $Z \perp\!\!\!\perp Y\{z, \text{clr}(\boldsymbol{m})\} \mid \boldsymbol{X}$;

Assumption S3 (No unmeasured $Z - \boldsymbol{M}$ confounding) For all z, $Z \perp\!\!\!\perp \text{clr}\{\boldsymbol{M}(z)\} \mid \boldsymbol{X}$;

Assumption S4 (No unmeasured $\boldsymbol{M} - Y$ confounding) For all $z, \boldsymbol{m}$, $\text{clr}(\boldsymbol{M}) \perp\!\!\!\perp Y\{z, \text{clr}(\boldsymbol{m})\} \mid \{Z, \boldsymbol{X}\}$;

Assumption S5 (No exposure-induced $\boldsymbol{M} - Y$ confounding) For all $\boldsymbol{m}$, $\text{clr}\{\boldsymbol{M}(0)\} \perp\!\!\!\perp Y\{1, \text{clr}(\boldsymbol{m})\} \mid \boldsymbol{X}$.

Assumption S4a (No unmeasured $\boldsymbol{M} - Y$ confounding) For all $z, \boldsymbol{m}$, $Y\{z, \text{clr}(\boldsymbol{m})\} \perp\!\!\!\perp \text{clr}(\boldsymbol{M}) \mid \{Z, L, \boldsymbol{X}\}$.

Assumption S6 (Positivity) For $z = 0, 1$ and all $\boldsymbol{x}$, $\Pr(Z = z \mid \boldsymbol{X} = \boldsymbol{x}) > 0$; for all $l, \boldsymbol{m}$, and $\boldsymbol{x}$, $E\{Y \mid Z = 0, L = l, \text{clr}(\boldsymbol{M}) = \text{clr}(\boldsymbol{m}), \boldsymbol{X} = \boldsymbol{x}\} > 0$; and for $z = 0, 1$ and all $l, \boldsymbol{m}$, and $\boldsymbol{x}$, $\Pr\{L = l \mid Z = z, \text{clr}(\boldsymbol{M}) = \text{clr}(\boldsymbol{m}), \boldsymbol{X} = \boldsymbol{x}\} > 0$.

Assumption S6a (Positivity) For $z = 0, 1$ and all $\boldsymbol{x}$, $\Pr(Z = z \mid \boldsymbol{X} = \boldsymbol{x}) > 0$; for all l, $\text{clr}(\boldsymbol{m}^{(1)})$, and $\boldsymbol{x}$, $E\{Y \mid Z = 0, L = l, \text{clr}(\boldsymbol{M}^{(1)}) = \text{clr}(\boldsymbol{m}^{(1)}), \boldsymbol{X} = \boldsymbol{x}\} > 0$; and for $z = 0, 1$ and all l, $\text{clr}(\boldsymbol{m}^{(1)})$, and $\boldsymbol{x}$, $\Pr\{L = l \mid Z = z, \text{clr}(\boldsymbol{M}^{(1)}) = \text{clr}(\boldsymbol{m}^{(1)}), \boldsymbol{X} = \boldsymbol{x}\} > 0$.

Assumption S6b (Positivity) For $z = 0, 1$ and all $\boldsymbol{x}$, $\Pr(Z = z \mid \boldsymbol{X} = \boldsymbol{x}) > 0$; and for all $\text{clr}(\boldsymbol{m}^{(1)})$ and $\boldsymbol{x}$, $E\{Y \mid Z = 0, \text{clr}(\boldsymbol{M}^{(1)}) = \text{clr}(\boldsymbol{m}^{(1)}), \boldsymbol{X} = \boldsymbol{x}\} > 0$.

S2.2. Identification of natural direct and indirect effects

Proposition S1 *(Mediation formula, Pearl, 2001) Suppose that Assumptions S1–S5 hold, then the NDE and NIE are identifiable. If $\boldsymbol{X}$ and $\text{clr}(\boldsymbol{M})$ are discrete, then*

$$\text{NDE} = \sum_{\text{clr}(\boldsymbol{m}), \boldsymbol{x}} \left[E\left\{Y \mid z_1, \text{clr}(\boldsymbol{m}), \boldsymbol{x}\right\} - E\left\{Y \mid z_0, \text{clr}(\boldsymbol{m}), \boldsymbol{x}\right\}\right] P\left\{\text{clr}(\boldsymbol{m}) \mid z_0, \boldsymbol{x}\right\} P(\boldsymbol{x}),$$

$$\text{NIE} = \sum_{\text{clr}(\boldsymbol{m}), \boldsymbol{x}} E\left\{Y \mid z_1, \text{clr}(\boldsymbol{m}), \boldsymbol{x}\right\} \left[P\left\{\text{clr}(\boldsymbol{m}) \mid z_1, \boldsymbol{x}\right\} - P\left\{\text{clr}(\boldsymbol{m}) \mid z_0, \boldsymbol{x}\right\}\right] P(\boldsymbol{x}),$$

where, following the standard shorthand in the mediation literature, $E\{Y \mid z_1, \text{clr}(\boldsymbol{m}), \boldsymbol{x}\} = E\{Y \mid Z = 1, \text{clr}(\boldsymbol{M}) = \text{clr}(\boldsymbol{m}), \boldsymbol{X} = \boldsymbol{x}\}$, $E\{Y \mid z_0, \text{clr}(\boldsymbol{m}), \boldsymbol{x}\} = E\{Y \mid Z = 0, \text{clr}(\boldsymbol{M}) = \text{clr}(\boldsymbol{m}), \boldsymbol{X} = \boldsymbol{x}\}$, $P\{\text{clr}(\boldsymbol{m}) \mid z_1, \boldsymbol{x}\} = \Pr\{\text{clr}(\boldsymbol{M}) = \text{clr}(\boldsymbol{m}) \mid Z = 1, \boldsymbol{X} = \boldsymbol{x}\}$, $P\{\text{clr}(\boldsymbol{m}) \mid z_0, \boldsymbol{x}\} = \Pr\{\text{clr}(\boldsymbol{M}) = \text{clr}(\boldsymbol{m}) \mid Z = 0, \boldsymbol{X} = \boldsymbol{x}\}$, and $P(\boldsymbol{x}) = \Pr(\boldsymbol{X} = \boldsymbol{x})$.

The proof of Proposition S1 is provided in Section S3.1. Under the following nonparametric structural equation models (NPSEM):

$$\boldsymbol{X} = f_{\boldsymbol{X}}(\boldsymbol{\epsilon}_{\boldsymbol{X}}),\ Z(\boldsymbol{x}) = f_Z(\boldsymbol{x}, \epsilon_Z),\ \text{clr}\{\boldsymbol{M}(\boldsymbol{x}, z)\} = f_{\text{clr}(\boldsymbol{M})}(\boldsymbol{x}, z, \boldsymbol{\epsilon}_{\boldsymbol{M}}),\ Y\{\boldsymbol{x}, z, \text{clr}(\boldsymbol{m})\} = f_Y\{\boldsymbol{x}, z, \text{clr}(\boldsymbol{m}), \epsilon_Y\}. \tag{S1}$$

Assumptions S2–S5 follow from the independent error (IE) assumption that $\boldsymbol{\epsilon}_{\boldsymbol{X}} \perp\!\!\!\perp \epsilon_Z \perp\!\!\!\perp \boldsymbol{\epsilon}_{\boldsymbol{M}} \perp\!\!\!\perp \epsilon_Y$. Supplementary Figure S1 shows the causal diagram corresponding to the NPSEM in (S1).

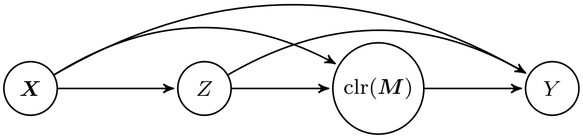

Supplementary Figure S1: A causal diagram corresponding to the nonparametric structural equation models (NPSEM) in (S1).

S2.3. Identification of interventional direct and indirect effects

Proposition S2 *(VanderWeele et al., 2014) Suppose that Assumptions S1 – S3 and S4a hold. Then IDE and IIE are identifiable. If $\boldsymbol{X}$, L, and $\text{clr}(\boldsymbol{M})$ are discrete, then*

$$\begin{aligned}\text{IDE} &= \sum_{l,\text{clr}(\boldsymbol{m}),\boldsymbol{x}} \left[E\{Y \mid z_1, l, \text{clr}(\boldsymbol{m}), \boldsymbol{x}\}P(l \mid z_1, \boldsymbol{x}) - E\{Y \mid z_0, l, \text{clr}(\boldsymbol{m}), \boldsymbol{x}\}P(l \mid z_0, \boldsymbol{x})\right] P\{\text{clr}(\boldsymbol{m}) \mid z_0, \boldsymbol{x}\}P(\boldsymbol{x}); \\ \text{IIE} &= \sum_{l,\text{clr}(\boldsymbol{m}),\boldsymbol{x}} E\{Y \mid z_1, l, \text{clr}(\boldsymbol{m}), \boldsymbol{x}\}P(l \mid z_1, \boldsymbol{x}) \left[P\{\text{clr}(\boldsymbol{m}) \mid z_1, \boldsymbol{x}\} - P\{\text{clr}(\boldsymbol{m}) \mid z_0, \boldsymbol{x}\}\right] P(\boldsymbol{x}),\end{aligned} \tag{S2}$$

where we use the shorthand that $E\{Y \mid z_1, l, \text{clr}(\boldsymbol{m}), \boldsymbol{x}\} = E\{Y \mid Z = 1, L = l, \text{clr}(\boldsymbol{M}) = \text{clr}(\boldsymbol{m}), \boldsymbol{X} = \boldsymbol{x}\}$, $E\{Y \mid z_0, l, \text{clr}(\boldsymbol{m}), \boldsymbol{x}\} = E\{Y \mid Z = 0, L = l, \text{clr}(\boldsymbol{M}) = \text{clr}(\boldsymbol{m}), \boldsymbol{X} = \boldsymbol{x}\}$, $P(l \mid z_1, \boldsymbol{x}) = \Pr(L = l \mid Z = 1, \boldsymbol{X} = \boldsymbol{x})$, $P(l \mid z_0, \boldsymbol{x}) = \Pr(L = l \mid Z = 0, \boldsymbol{X} = \boldsymbol{x})$, and other shorthand notation follows Proposition S1.

The proof of Proposition S2 is provided in Section S3.2. The identification expression in (S2) naturally extends to continuous $\boldsymbol{X}$, L, and $\text{clr}(\boldsymbol{M})$ by replacing summation with integration. Assumptions S2, S3, and S4a are satisfied under the causal diagram in Figure 1 of the main manuscript; they would also hold if the causal association between L and Y were confounded by additional unmeasured factors.

Remark S1 *Assumption S5 is a "cross-world" independence assumption (Robins and Richardson, 2010), meaning that it cannot be validated by any randomized experiment involving the variables in Figure S1. In contrast, all the assumptions required for Proposition S2 are "single-world" and can be guaranteed under randomization of both Z and $\text{clr}(\boldsymbol{M})$.*

Remark S2 *If L is absent, then the identification formulas for the IDE and IIE reduce to those for the NDE and NIE.*

S2.4. Algorithm for estimating the IIE

Algorithm S1 Proposed inverse probability weighting approach for estimating the IIE

1. Fit logistic regression models for $\Pr(Z = 1 \mid \boldsymbol{X}; \boldsymbol{\alpha})$ and $\Pr(L = 1 \mid Z, \boldsymbol{X}; \boldsymbol{\gamma})$ using the maximum likelihood estimation. Let $\hat{\Pr}(Z = z \mid \boldsymbol{X}) = \Pr(Z = z \mid \boldsymbol{X}; \hat{\boldsymbol{\alpha}})$ and $\hat{\Pr}(L \mid Z = z, \boldsymbol{X}) = \Pr(L \mid Z = z, \boldsymbol{X}; \hat{\boldsymbol{\gamma}}), z \in \{0, 1\}$, where $\hat{\boldsymbol{\alpha}}$ and $\hat{\boldsymbol{\gamma}}$ denote the maximum likelihood estimates of $\boldsymbol{\alpha}$ and $\boldsymbol{\gamma}$, respectively.
2. Estimate $E(Y \mid Z = z, L, \text{clr}(\boldsymbol{M}^{(1)}), \boldsymbol{X})$, $z = 0, 1$, using the penalized logistic regression procedure described earlier. Let $\text{clr}(\hat{\boldsymbol{M}}^{(1)})$ denote the selected set of mediators and $\hat{E}(Y \mid Z = z, L, \text{clr}(\hat{\boldsymbol{M}}^{(1)}), \boldsymbol{X})$ the corresponding fitted conditional means.
3. Estimate $\Pr(L \mid Z = z, \text{clr}(\hat{\boldsymbol{M}}^{(1)}), \boldsymbol{X}), z = 0, 1$, using bagging with the optimal subset of DNNs. Let $\hat{\Pr}(L \mid Z = z, \text{clr}(\hat{\boldsymbol{M}}^{(1)}), \boldsymbol{X})$ denote the corresponding fitted conditional probabilities.
4. The estimator of the IIE is

$$\begin{aligned}\hat{\text{IIE}} = \mathbb{P}_n &\left\{ \frac{ZY}{\hat{\Pr}(Z = 1 \mid \boldsymbol{X})} \frac{\hat{\Pr}(L \mid Z = 1, \boldsymbol{X})}{\hat{\Pr}(L \mid Z = 1, \text{clr}(\hat{\boldsymbol{M}}^{(1)}), \boldsymbol{X})} \right\} \\ &- \mathbb{P}_n \left\{ \frac{(1 - Z)Y}{\hat{\Pr}(Z = 0 \mid \boldsymbol{X})} \frac{\hat{E}(Y \mid Z = 1, L, \text{clr}(\hat{\boldsymbol{M}}^{(1)}), \boldsymbol{X})\hat{\Pr}(L \mid Z = 1, \boldsymbol{X})}{\hat{E}(Y \mid Z = 0, L, \text{clr}(\hat{\boldsymbol{M}}^{(1)}), \boldsymbol{X})\hat{\Pr}(L \mid Z = 0, \text{clr}(\hat{\boldsymbol{M}}^{(1)}), \boldsymbol{X})} \right\},\end{aligned}$$

where $\mathbb{P}_n$ denotes the empirical mean operator.

S2.5. The implementation details of bagging with the optimal subset of deep neural networks

To implement bagging with the optimal subset of deep neural networks (DNNs), we follow the approach of Mi et al. (2019) and use their R package `deepTL`. Documentation for `deepTL` is available at `https://github.com/SkadiEye/deepTL`.

In both the simulation studies and real data analysis, we use fully connected feedforward DNNs with one hidden layer containing five hidden nodes. The activation function is the rectified linear unit (ReLU), defined as $\text{ReLU}(t) = \max(0, t)$. The tuning parameter λ for the L_1 penalty function is set to

10^{-5}. Mini-batch stochastic gradient descent is performed using a batch size of 20 for a maximum of 100 training epochs, with Adam used as the adaptive optimization algorithm (Kingma and Ba, 2014). We train an ensemble of 20 DNNs, and the final ensemble is automatically selected according to the criterion proposed by Mi et al. (2019). The simulation results are found to be robust to reasonable choices of these tuning parameters.

S2.6. Rationale underlying the proposed data-generation mechanism for the clr-transformed mediators

Recall that, in Section 3.1 of the main manuscript, we generate the clr-transformed mediators directly rather than simulating microbiome read counts. This strategy substantially reduces the computational burden of evaluating the true interventional indirect effect while retaining selected empirical characteristics of the observed AML microbiome data relevant to the simulation design. Here we describe the rationale underlying the proposed data-generation mechanism.

In the AML microbiome study, microbiome measurements are recorded as read counts. Let R_{ik} denote the observed read count of the kth taxon for the ith sample, where $i \in \{1, \ldots, n\}$ and $k \in \{1, \ldots, q\}$. Let

$$M_{ik} = \frac{R_{ik}}{\sum_{j=1}^{q} R_{ij}}$$

denote the corresponding compositional relative abundance. Thus, each relative abundance is defined relative to the total read count across all taxa.

It is useful to distinguish exposure effects on the underlying absolute-abundance scale from those on the compositional relative-abundance and clr-transformed scales. From a biological perspective, an exposure may directly affect the underlying absolute abundances of only a subset of microbial taxa. However, such sparsity does not generally carry over to the compositional relative-abundance scale. Because each relative abundance is defined with respect to the total abundance across all taxa, changes in a subset of taxa can change the common denominator and consequently alter the relative abundances of taxa whose underlying absolute abundances are not directly affected.

The clr transformation further expresses each taxon's relative abundance as the log-ratio of that relative abundance to the geometric mean of the relative abundances across all taxa:

$$\operatorname{clr}(M_{ik}) = \log\left\{\frac{M_{ik}}{g(\boldsymbol{M}_i)}\right\}, \qquad g(\boldsymbol{M}_i) = \left(\prod_{j=1}^{q} M_{ij}\right)^{1/q}.$$

Thus, the clr-transformed value of taxon k depends not only on its own relative abundance M_{ik}, but also on the relative abundances of all other taxa through the common geometric mean $g(\boldsymbol{M}_i)$. Consequently, when the underlying absolute abundances of a subset of taxa change, the resulting changes in the compositional relative abundances can alter $g(\boldsymbol{M}_i)$, which in turn can change the clr-transformed values corresponding to taxa whose absolute abundances are not directly affected. Therefore, sparsity on the underlying absolute-abundance scale does not generally translate into sparsity on the clr-transformed scale.

We next describe how the empirical characteristics of the AML microbiome data motivate our direct generation of the clr-transformed mediators. Because microbiome read counts are highly zero-inflated, zero counts are replaced by a small positive constant δ (e.g., $\delta = 0.5$) before calculating the relative abundances and applying the clr transformation. Let

$$\widetilde{R}_{ik} = \begin{cases} R_{ik}, & R_{ik} > 0, \\ \delta, & R_{ik} = 0, \end{cases} \qquad \widetilde{T}_i = \sum_{k=1}^{q} \widetilde{R}_{ik},$$

and define

$$\widetilde{M}_{ik} = \frac{\widetilde{R}_{ik}}{\widetilde{T}_i}.$$

For a taxon with an original zero read count, $\widetilde{R}_{ik} = \delta$, and its clr-transformed value is

$$\operatorname{clr}\left(\frac{\delta}{\widetilde{T}_i}\right) = \log\left(\frac{\delta}{\widetilde{T}_i}\right) - \frac{1}{q}\sum_{k=1}^{q} \log\left(\frac{\widetilde{R}_{ik}}{\widetilde{T}_i}\right) \triangleq B_i.$$

Since $\widetilde{R}_{ik} \geq \delta$ for all taxa and $\widetilde{R}_{ik} > \delta$ for at least one taxon, we have $B_i < 0$. Define $C_i = -B_i$, so that $C_i > 0$.

Supplementary Figure S2 shows that the observed values of C_i in the AML microbiome study are positive and right-skewed. Motivated by this empirical feature, we assume

$$C_i \mid (Z_i, L_i, \boldsymbol{X}_i) \sim \operatorname{Gamma}\{\eta_0, \theta(Z_i, L_i, \boldsymbol{X}_i)\},$$

where η_0 is the shape parameter and

$$\theta(Z_i, L_i, \boldsymbol{X}_i) = \frac{\exp\left(\theta_0 + \theta_Z Z_i + \theta_L L_i + \boldsymbol{\theta}_{\boldsymbol{X}}^\top \boldsymbol{X}_i\right)}{\eta_0}$$

is the scale parameter. Under this parameterization,

$$E(C_i \mid Z_i, L_i, \boldsymbol{X}_i) = \exp\left(\theta_0 + \theta_Z Z_i + \theta_L L_i + \boldsymbol{\theta}_{\boldsymbol{X}}^\top \boldsymbol{X}_i\right),$$

and

$$\operatorname{Var}(C_i \mid Z_i, L_i, \boldsymbol{X}_i) = \frac{\{E(C_i \mid Z_i, L_i, \boldsymbol{X}_i)\}^2}{\eta_0},$$

corresponding to a Gamma generalized linear model with a log link.

Under this data-generation mechanism, θ_Z controls whether Z directly affects the distribution of the clr-transformed mediators through C_i, conditional on L_i and $\boldsymbol{X}_i$. It does not parameterize how many taxa are directly affected by the exposure on the underlying absolute-abundance scale.

To evaluate the Gamma generalized linear modeling assumption for C_i, we fit the corresponding Gamma generalized linear model to the AML data and assess its adequacy using randomized quantile residual diagnostics implemented in the R package `DHARMa`. The Kolmogorov–Smirnov test (p-value $= 0.55$), dispersion test (p-value $= 0.60$), and outlier test (p-value $= 1.00$) provide no evidence of lack of fit, overdispersion, or excessive outliers, supporting the use of the Gamma generalized linear model for the conditional distribution of C_i.

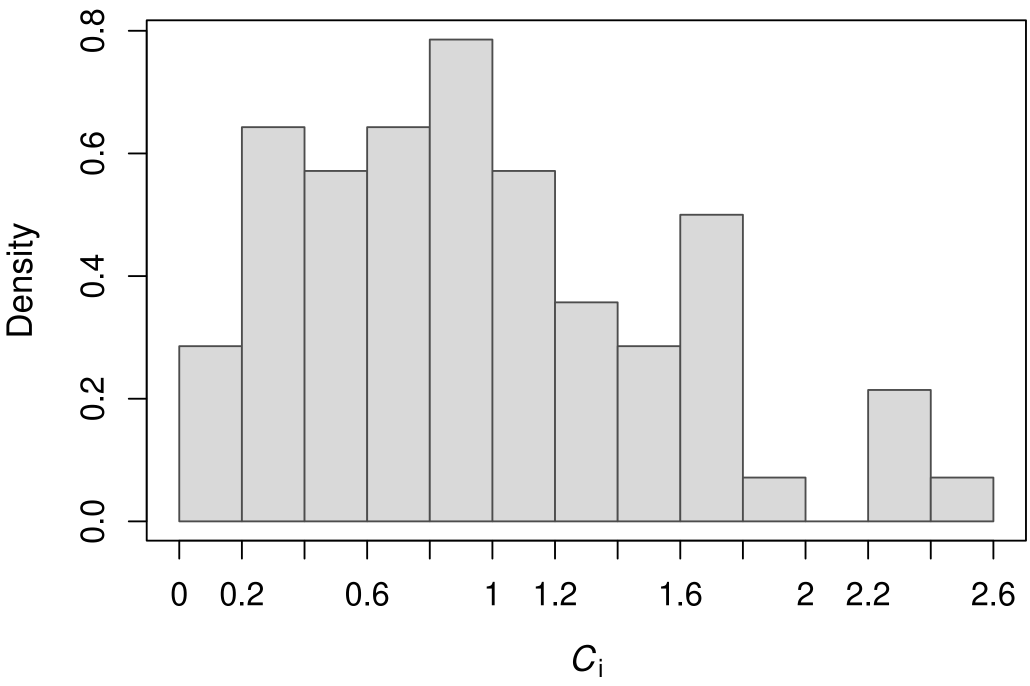

Supplementary Figure S2: Histogram of the observed values of C_i in the AML microbiome study plotted on the density scale.

For sample i, let ζ_i denote the number of taxa among the first $q-1$ components that have zero read counts before zero replacement and therefore take the clr-transformed value $B_i = -C_i$. Examination of the AML microbiome data indicates that the distribution of ζ_i is reasonably approximated by a discrete uniform distribution. We therefore generate

$$\zeta_i \sim \mathrm{DiscreteUniform}\left\{\lfloor (q-1)a \rfloor, \ldots, \lfloor (q-1)b \rfloor\right\},$$

where $\lfloor \cdot \rfloor$ denotes the floor function. The constants a and b determine the range of zero-count proportions in the simulated microbiome data. In our simulation studies, we set $a = 0.4$ and $b = 0.8$, allowing the proportion of zero read counts to range from moderate to high levels while remaining representative of the zero inflation observed in the AML microbiome study.

Conditional on ζ_i, exactly ζ_i of the first $q-1$ clr-transformed mediators take the value $B_i = -C_i$. The remaining $q-1-\zeta_i$ components are assumed, for simplicity, to follow a uniform distribution conditional on C_i and ζ_i. Denote these components by

$$\left\{\mathrm{clrM}_{i(1)}, \ldots, \mathrm{clrM}_{i(q-1-\zeta_i)}\right\}.$$

Because clr-transformed components satisfy the sum-to-zero constraint,

$$\mathrm{clrM}_{iq} = -\sum_{k=1}^{q-1} \mathrm{clrM}_{ik}.$$

In addition, $B_i = -C_i$ is the clr-transformed value corresponding to a zero read count, so we require

$$\mathrm{clrM}_{iq} \geq B_i.$$

Since ζ_i of the first $q-1$ components equal B_i, this requirement implies

$$-\left\{\zeta_i B_i + \sum_{s=1}^{q-1-\zeta_i} \mathrm{clrM}_{i(s)}\right\} \geq B_i,$$

or equivalently,

$$\sum_{s=1}^{q-1-\zeta_i} \mathrm{clrM}_{i(s)} \leq -(1+\zeta_i)B_i = (1+\zeta_i)C_i.$$

A sufficient condition for this constraint is

$$\mathrm{clrM}_{i(s)} \leq \frac{(1+\zeta_i)C_i}{q-1-\zeta_i}, \qquad s = 1, \ldots, q-1-\zeta_i,$$

which determines the upper endpoint of the uniform distribution.

To center the marginal distribution of each of the first $q-1$ clr-transformed mediators, we choose the lower endpoint a_i such that

$$E(\mathrm{clrM}_{ik} \mid C_i, \zeta_i) = 0, \qquad k = 1, \ldots, q-1.$$

This yields

$$a_i = -\frac{C_i(1-\zeta_i)}{q-1-\zeta_i}.$$

Under random assignment of the ζ_i zero components among the first $q-1$ taxa, the conditional distribution of each of these clr-transformed mediators is

$$\mathrm{clr}M_{ik} \mid C_i, \zeta_i \sim \frac{\zeta_i}{q-1}\,\mathrm{Dirac}(-C_i) + \left(1 - \frac{\zeta_i}{q-1}\right) \mathrm{Uniform}\left(a_i, \frac{(1+\zeta_i)C_i}{q-1-\zeta_i}\right), \qquad k = 1, \ldots, q-1,$$

where $\mathrm{Dirac}(-C_i)$ denotes the point-mass distribution at $-C_i$. It is straightforward to verify that

$$a_i \geq -C_i$$

for $q \geq 2$ and $0 \leq \zeta_i \leq q-2$, and

$$a_i < \frac{(1+\zeta_i)C_i}{q-1-\zeta_i},$$

so the support of the uniform distribution is well defined. Finally, setting

$$\mathrm{clrM}_{iq} = -\sum_{k=1}^{q-1} \mathrm{clrM}_{ik}$$

ensures that the simulated clr-transformed mediator vector satisfies the inherent sum-to-zero constraint, and $E(\mathrm{clrM}_{iq}) = 0$.

S2.7. Estimation and hypothesis testing methods for the natural effects

Following the same idea as the proposed estimation method for the interventional effects, the natural direct effect (NDE) and natural indirect effect (NIE) can be estimated using identification formulas that avoid modeling the conditional distributions of $\mathrm{clr}(\boldsymbol{M})$. The result is given in Proposition S3.

Proposition S3 *Suppose that there exist subsets* $\mathrm{clr}(\boldsymbol{M}^{(1)})$ *and* $\mathrm{clr}(\boldsymbol{M}^{(2)})$ *of the components of* $\mathrm{clr}(\boldsymbol{M})$ *such that* $\mathrm{clr}(\boldsymbol{M}^{(1)}) \cup \mathrm{clr}(\boldsymbol{M}^{(2)}) = \mathrm{clr}(\boldsymbol{M})$*,* $\mathrm{clr}(\boldsymbol{M}^{(1)}) \cap \mathrm{clr}(\boldsymbol{M}^{(2)}) = \emptyset$*,* $\mathrm{clr}(\boldsymbol{M}^{(1)}) \not\perp\!\!\!\perp Y \mid \{Z, \boldsymbol{X}\}$*, and* $\mathrm{clr}(\boldsymbol{M}^{(2)}) \perp\!\!\!\perp Y \mid \{Z, \boldsymbol{X}, \mathrm{clr}(\boldsymbol{M}^{(1)})\}$*. Both subsets retain the clr values computed from the full composition* $\boldsymbol{M}$*. Under Assumptions S1–S5 and S6b, NDE and NIE are identified as*

$$\mathrm{NDE} = E\left\{\frac{(1-Z)Y}{\Pr(Z=0 \mid \boldsymbol{X})} \frac{E(Y \mid Z=1, \mathrm{clr}(\boldsymbol{M}^{(1)}), \boldsymbol{X})}{E(Y \mid Z=0, \mathrm{clr}(\boldsymbol{M}^{(1)}), \boldsymbol{X})}\right\} - E\left\{\frac{(1-Z)Y}{\Pr(Z=0 \mid \boldsymbol{X})}\right\},$$

$$\mathrm{NIE} = E\left\{\frac{ZY}{\Pr(Z=1 \mid \boldsymbol{X})}\right\} - E\left\{\frac{(1-Z)Y}{\Pr(Z=0 \mid \boldsymbol{X})} \frac{E(Y \mid Z=1, \mathrm{clr}(\boldsymbol{M}^{(1)}), \boldsymbol{X})}{E(Y \mid Z=0, \mathrm{clr}(\boldsymbol{M}^{(1)}), \boldsymbol{X})}\right\}.$$

Based on Proposition S3, estimation of the NDE and NIE proceeds analogously to the procedure in Section 2.2.3 in the main manuscript, with two key differences: (i) modeling of the exposure-induced mediator–outcome confounder L is not required; and (ii) we estimate $E(Y \mid Z = z, \mathrm{clr}(\boldsymbol{M}^{(1)}), \boldsymbol{X})$ for $z = 0, 1$ instead of the conditional expectations involving L. These conditional expectations can be obtained using penalized logistic regression, following the same strategy described in Section 2.2.3 in the main manuscript but omitting L from the outcome model. Hypothesis testing for the mediation effect can be performed by testing

$$H_0 : \mathrm{NIE} = 0 \quad \text{versus} \quad H_a : \mathrm{NIE} \neq 0,$$

using the same bootstrap confidence interval (CI) approach described in Section 2.2.4 in the main manuscript for the IIE.

We refer to the estimation and testing procedures for the NDE as the IPW-NDE method, and the corresponding procedures for the NIE as the IPW-NIE method.

S2.8. Comparison of three bootstrap CIs in the simulation studies

Note that the type-I error rates and power reported for the proposed method and the IPW-NIE method in Tables 1 and 2 of the main manuscript are based on the standard normal bootstrap CI (denoted as Normal CI), following the procedure described in Section 2.2.4 of the main manuscript and Supplementary Note S2.7. To compare the performance of different bootstrap CIs, we additionally consider the bootstrap percentile CI (denoted as Percentile CI; Efron and Tibshirani (1994)) and the bias-corrected and accelerated bootstrap CI (denoted as BCa CI; Efron (1987)). The coverage probabilities of the resulting 95% CIs are presented in Supplementary Tables S2 and S3, and the corresponding power results under nonzero mediation effect scenarios are reported in Supplementary Table S4.

Under zero mediation effect scenarios (Supplementary Table S2), the type-I error rate is equal to one minus the coverage probability. For the proposed method, the coverage probabilities of the Normal CI are generally close to the nominal level of 0.95, implying type-I error rates close to the nominal significance level of 0.05. The Percentile CI and BCa CI often exhibit higher coverage probabilities than the Normal CI and, in several scenarios, yield coverage probabilities close to one, indicating more conservative inference and correspondingly lower type-I error rates. Similar patterns are observed for the IPW-NIE method when the NIE is identifiable ($\gamma_Z = \theta_Z = 0$). However, when the NIE is not identifiable ($\theta_Z \neq 0$), all three CIs for the IPW-NIE method exhibit substantial departures from the nominal level, regardless of the bootstrap procedure used.

Under nonzero mediation effect scenarios (Supplementary Tables S3 and S4), the Normal CI for the proposed method generally achieves coverage probabilities close to the nominal level while often providing higher power than the Percentile CI and BCa CI, particularly when the sample size is small ($n = 70$). As the sample size increases to $n = 200$, the power differences among the three CI procedures become less pronounced, although the Percentile CI and BCa CI often exhibit slightly higher coverage probabilities than the Normal CI. Similar patterns are observed for the IPW-NIE method. In general, the coverage probabilities of the Normal CI for the IPW-NIE method exhibit larger departures from the nominal level than those of the proposed method.

Overall, the Normal CI used by the proposed method achieves coverage probabilities closest to the nominal level across the scenarios considered while maintaining appropriate type-I error control and often providing higher power than the more conservative Percentile CI and BCa CI. Taken together, these results suggest that the Normal CI provides a favorable balance between coverage accuracy and statistical power for the proposed method.

S2.9. Computational cost for the AML microbiome data analysis

Regarding computational cost, the real-data analysis was performed on a 40-core node equipped with an Intel Cascade Lake processor, with all 40 cores used for parallel computing. All reported wall-clock times include the bootstrap calculations used to construct the CIs. For the analysis reported in Table 3 of the main manuscript, the proposed method required 3.45 seconds to obtain the IIE and IDE estimates and their corresponding CIs. The IPW-NIE and IPW-NDE methods required 2.19 seconds, whereas the Sohn-NIE and Sohn-NDE methods required substantially longer, with a total wall-clock time of 1,968.07 seconds.

S2.10. Biological context for the selected candidate mediators

The penalized logistic regression outcome model selected 11 clr-transformed microbial features from the genus-level abundance table as candidate mediators (Supplementary Table S5). Several selected features map to taxonomic groups containing members previously reported to change during chemotherapy-based cancer treatment, including Streptococcaceae, Bacteroidaceae, Enterobacteriaceae, Lachnospiraceae, Actinomycetaceae, Ruminococcaceae, Verrucomicrobiaceae, Rikenellaceae, Clostridiales, and Methanobacteriaceae (D'Amico et al., 2021; Papanicolas et al., 2021; Terrisse et al., 2021; Zhang et al., 2021; Wu et al., 2022; Xu et al., 2024). For example, Xu et al. (2024) reported that the abundance of Enterobacteriaceae decreased significantly after R-CHOP-based treatment in patients with diffuse large B-cell lymphoma. In a longitudinal study of patients with breast cancer, Wu et al. (2022) reported chemotherapy-regimen-associated abundance changes involving *Actinomyces* (family Actinomycetaceae), Rikenellaceae, *Akkermansia* (family Verrucomicrobiaceae), and *Methanobrevibacter* (family Methanobacteriaceae).

Several of these taxonomic groups have additionally been associated with infection or infection-related outcomes (Vincent et al., 2013; Khanna et al., 2016; Sangster et al., 2016; Hakim et al., 2018; Vázquez-Cuesta et al., 2023; McMahon et al., 2025). For example, Vincent et al. (2013) found that lower relative abundances of Bacteroidaceae and a family-level group within Clostridiales in the gut microbiome were associated with subsequent nosocomial *Clostridioides difficile* infection. These previously reported associations provide biological context for the selected features but should not be interpreted as independent confirmation of taxon-specific causal mediation effects.

S2.11. Sensitivity to zero-count replacement and compositional data transformation

To assess sensitivity to zero-count replacement before the clr transformation, we repeat the proposed mediation analysis using replacement values of 0.1, 0.5, and 0.9, where 0.5 is used in the primary analysis. These values allow us to examine the sensitivity of the results over a range of replacement values below the minimum nonzero count of 1 observed in the AML microbiome data. As shown in Supplementary Table S7, the estimated IIEs range from 0.403 to 0.443, and all corresponding 95% bootstrap CIs exclude zero. The estimated IDEs range from -0.115 to -0.063, with all CIs including zero. These results indicate that both the effect estimates and inferential conclusions remain stable across the replacement values examined.

We also assess sensitivity to the compositional data transformation by repeating the analysis using the additive log-ratio (alr) and isometric log-ratio (ilr) transformations in addition to the primary clr transformation (Lin and Peddada, 2020). As shown in Supplementary Table S8, the estimated IIEs are 0.403, 0.403, and 0.400 under the clr, alr, and ilr transformations, respectively, and all corresponding 95% bootstrap CIs exclude zero. The IDE CIs include zero under all three transformations. Thus, the conclusion of a significant IIE but a nonsignificant IDE remains unchanged across the log-ratio transformations considered.

S3. Supplementary Proofs

S3.1. Proof of Proposition S1

Proof If Assumptions S1–S5 hold, then

$$\begin{aligned}
\text{NDE} =& E\left[Y\left\{1, \text{clr}\left(\boldsymbol{M}(0)\right)\right\}\right] - E\left[Y\left\{0, \text{clr}\left(\boldsymbol{M}(0)\right)\right\}\right] \\
=& \sum_{\text{clr}(\boldsymbol{m}),\boldsymbol{x}} E[Y\{1, \text{clr}(\boldsymbol{m})\} \mid \text{clr}\{\boldsymbol{M}(0)\} = \text{clr}(\boldsymbol{m}), \boldsymbol{X} = \boldsymbol{x}] \Pr[\text{clr}\{\boldsymbol{M}(0)\} = \text{clr}(\boldsymbol{m}) \mid \boldsymbol{X} = \boldsymbol{x}] \Pr(\boldsymbol{X} = \boldsymbol{x}) \\
&- \sum_{\text{clr}(\boldsymbol{m}),\boldsymbol{x}} E[Y\{0, \text{clr}(\boldsymbol{m})\} \mid \text{clr}\{\boldsymbol{M}(0)\} = \text{clr}(\boldsymbol{m}), \boldsymbol{X} = \boldsymbol{x}] \Pr[\text{clr}\{\boldsymbol{M}(0)\} = \text{clr}(\boldsymbol{m}) \mid \boldsymbol{X} = \boldsymbol{x}] \Pr(\boldsymbol{X} = \boldsymbol{x}) \\
=& \sum_{\text{clr}(\boldsymbol{m}),\boldsymbol{x}} E[Y\{1, \text{clr}(\boldsymbol{m})\} \mid \boldsymbol{X} = \boldsymbol{x}] \Pr[\text{clr}\{\boldsymbol{M}(0)\} = \text{clr}(\boldsymbol{m}) \mid \boldsymbol{X} = \boldsymbol{x}] \Pr(\boldsymbol{X} = \boldsymbol{x}) \\
&- \sum_{\text{clr}(\boldsymbol{m}),\boldsymbol{x}} E[Y\{0, \text{clr}(\boldsymbol{m})\} \mid \boldsymbol{X} = \boldsymbol{x}] \Pr[\text{clr}\{\boldsymbol{M}(0)\} = \text{clr}(\boldsymbol{m}) \mid \boldsymbol{X} = \boldsymbol{x}] \Pr(\boldsymbol{X} = \boldsymbol{x}) \\
=& \sum_{\text{clr}(\boldsymbol{m}),\boldsymbol{x}} E[Y\{1, \text{clr}(\boldsymbol{m})\} \mid Z = 1, \boldsymbol{X} = \boldsymbol{x}] \Pr[\text{clr}\{\boldsymbol{M}(0)\} = \text{clr}(\boldsymbol{m}) \mid Z = 0, \boldsymbol{X} = \boldsymbol{x}] \Pr(\boldsymbol{X} = \boldsymbol{x}) \\
&- \sum_{\text{clr}(\boldsymbol{m}),\boldsymbol{x}} E[Y\{0, \text{clr}(\boldsymbol{m})\} \mid Z = 0, \boldsymbol{X} = \boldsymbol{x}] \Pr[\text{clr}\{\boldsymbol{M}(0)\} = \text{clr}(\boldsymbol{m}) \mid Z = 0, \boldsymbol{X} = \boldsymbol{x}] \Pr(\boldsymbol{X} = \boldsymbol{x}) \\
=& \sum_{\text{clr}(\boldsymbol{m}),\boldsymbol{x}} E[Y\{1, \text{clr}(\boldsymbol{m})\} \mid Z = 1, \text{clr}(\boldsymbol{M}) = \text{clr}(\boldsymbol{m}), \boldsymbol{X} = \boldsymbol{x}] \Pr[\text{clr}\{\boldsymbol{M}(0)\} = \text{clr}(\boldsymbol{m}) \mid Z = 0, \boldsymbol{X} = \boldsymbol{x}] \Pr(\boldsymbol{X} = \boldsymbol{x}) \\
&- \sum_{\text{clr}(\boldsymbol{m}),\boldsymbol{x}} E[Y\{0, \text{clr}(\boldsymbol{m})\} \mid Z = 0, \text{clr}(\boldsymbol{M}) = \text{clr}(\boldsymbol{m}), \boldsymbol{X} = \boldsymbol{x}] \Pr[\text{clr}\{\boldsymbol{M}(0)\} = \text{clr}(\boldsymbol{m}) \mid Z = 0, \boldsymbol{X} = \boldsymbol{x}] \Pr(\boldsymbol{X} = \boldsymbol{x}) \\
=& \sum_{\text{clr}(\boldsymbol{m}),\boldsymbol{x}} E\{Y \mid Z = 1, \text{clr}(\boldsymbol{M}) = \text{clr}(\boldsymbol{m}), \boldsymbol{X} = \boldsymbol{x}\} \Pr\{\text{clr}(\boldsymbol{M}) = \text{clr}(\boldsymbol{m}) \mid Z = 0, \boldsymbol{X} = \boldsymbol{x}\} \Pr(\boldsymbol{X} = \boldsymbol{x}) \\
&- \sum_{\text{clr}(\boldsymbol{m}),\boldsymbol{x}} E\{Y \mid Z = 0, \text{clr}(\boldsymbol{M}) = \text{clr}(\boldsymbol{m}), \boldsymbol{X} = \boldsymbol{x}\} \Pr\{\text{clr}(\boldsymbol{M}) = \text{clr}(\boldsymbol{m}) \mid Z = 0, \boldsymbol{X} = \boldsymbol{x}\} \Pr(\boldsymbol{X} = \boldsymbol{x}) \\
=& \sum_{\text{clr}(\boldsymbol{m}),\boldsymbol{x}} \Big[\{E\left(Y \mid Z = 1, \text{clr}(\boldsymbol{M}) = \text{clr}(\boldsymbol{m}), \boldsymbol{X} = \boldsymbol{x}\right) - E\left(Y \mid Z = 0, \text{clr}(\boldsymbol{M}) = \text{clr}(\boldsymbol{m}), \boldsymbol{X} = \boldsymbol{x}\right)\} \\
&\quad \times \Pr\{\text{clr}(\boldsymbol{M}) = \text{clr}(\boldsymbol{m}) \mid Z = 0, \boldsymbol{X} = \boldsymbol{x}\} \Pr(\boldsymbol{X} = \boldsymbol{x})\Big].
\end{aligned}$$

$$
\begin{aligned}
\mathrm{NIE} =& E\left[Y\left\{1, \operatorname{clr}\left(\boldsymbol{M}(1)\right)\right\}\right]-E\left[Y\left\{1, \operatorname{clr}\left(\boldsymbol{M}(0)\right)\right\}\right] \\
=& \sum_{\operatorname{clr}(\boldsymbol{m}), \boldsymbol{x}} E[Y\{1, \operatorname{clr}(\boldsymbol{m})\} \mid \operatorname{clr}\{\boldsymbol{M}(1)\}=\operatorname{clr}(\boldsymbol{m}), \boldsymbol{X}=\boldsymbol{x}] \operatorname{Pr}[\operatorname{clr}\{\boldsymbol{M}(1)\}=\operatorname{clr}(\boldsymbol{m}) \mid \boldsymbol{X}=\boldsymbol{x}] \operatorname{Pr}(\boldsymbol{X}=\boldsymbol{x}) \\
& -\sum_{\operatorname{clr}(\boldsymbol{m}), \boldsymbol{x}} E[Y\{1, \operatorname{clr}(\boldsymbol{m})\} \mid \operatorname{clr}\{\boldsymbol{M}(0)\}=\operatorname{clr}(\boldsymbol{m}), \boldsymbol{X}=\boldsymbol{x}] \operatorname{Pr}[\operatorname{clr}\{\boldsymbol{M}(0)\}=\operatorname{clr}(\boldsymbol{m}) \mid \boldsymbol{X}=\boldsymbol{x}] \operatorname{Pr}(\boldsymbol{X}=\boldsymbol{x}) \\
=& \sum_{\operatorname{clr}(\boldsymbol{m}), \boldsymbol{x}} E[Y\{1, \operatorname{clr}(\boldsymbol{m})\} \mid \boldsymbol{X}=\boldsymbol{x}] \operatorname{Pr}[\operatorname{clr}\{\boldsymbol{M}(1)\}=\operatorname{clr}(\boldsymbol{m}) \mid \boldsymbol{X}=\boldsymbol{x}] \operatorname{Pr}(\boldsymbol{X}=\boldsymbol{x}) \\
& -\sum_{\operatorname{clr}(\boldsymbol{m}), \boldsymbol{x}} E[Y\{1, \operatorname{clr}(\boldsymbol{m})\} \mid \boldsymbol{X}=\boldsymbol{x}] \operatorname{Pr}[\operatorname{clr}\{\boldsymbol{M}(0)\}=\operatorname{clr}(\boldsymbol{m}) \mid \boldsymbol{X}=\boldsymbol{x}] \operatorname{Pr}(\boldsymbol{X}=\boldsymbol{x}) \\
=& \sum_{\operatorname{clr}(\boldsymbol{m}), \boldsymbol{x}} E[Y\{1, \operatorname{clr}(\boldsymbol{m})\} \mid Z=1, \boldsymbol{X}=\boldsymbol{x}] \operatorname{Pr}[\operatorname{clr}\{\boldsymbol{M}(1)\}=\operatorname{clr}(\boldsymbol{m}) \mid Z=1, \boldsymbol{X}=\boldsymbol{x}] \operatorname{Pr}(\boldsymbol{X}=\boldsymbol{x}) \\
& -\sum_{\operatorname{clr}(\boldsymbol{m}), \boldsymbol{x}} E[Y\{1, \operatorname{clr}(\boldsymbol{m})\} \mid Z=1, \boldsymbol{X}=\boldsymbol{x}] \operatorname{Pr}[\operatorname{clr}\{\boldsymbol{M}(0)\}=\operatorname{clr}(\boldsymbol{m}) \mid Z=0, \boldsymbol{X}=\boldsymbol{x}] \operatorname{Pr}(\boldsymbol{X}=\boldsymbol{x}) \\
=& \sum_{\operatorname{clr}(\boldsymbol{m}), \boldsymbol{x}} E[Y\{1, \operatorname{clr}(\boldsymbol{m})\} \mid Z=1, \operatorname{clr}(\boldsymbol{M})=\operatorname{clr}(\boldsymbol{m}), \boldsymbol{X}=\boldsymbol{x}] \operatorname{Pr}[\operatorname{clr}\{\boldsymbol{M}(1)\}=\operatorname{clr}(\boldsymbol{m}) \mid Z=1, \boldsymbol{X}=\boldsymbol{x}] \operatorname{Pr}(\boldsymbol{X}=\boldsymbol{x}) \\
-& \sum_{\operatorname{clr}(\boldsymbol{m}), \boldsymbol{x}} E[Y\{1, \operatorname{clr}(\boldsymbol{m})\} \mid Z=1, \operatorname{clr}(\boldsymbol{M})=\operatorname{clr}(\boldsymbol{m}), \boldsymbol{X}=\boldsymbol{x}] \operatorname{Pr}[\operatorname{clr}\{\boldsymbol{M}(0)\}=\operatorname{clr}(\boldsymbol{m}) \mid Z=0, \boldsymbol{X}=\boldsymbol{x}] \operatorname{Pr}(\boldsymbol{X}=\boldsymbol{x}) \\
=& \sum_{\operatorname{clr}(\boldsymbol{m}), \boldsymbol{x}} E\{Y \mid Z=1, \operatorname{clr}(\boldsymbol{M})=\operatorname{clr}(\boldsymbol{m}), \boldsymbol{X}=\boldsymbol{x}\} \operatorname{Pr}\{\operatorname{clr}(\boldsymbol{M})=\operatorname{clr}(\boldsymbol{m}) \mid Z=1, \boldsymbol{X}=\boldsymbol{x}\} \operatorname{Pr}(\boldsymbol{X}=\boldsymbol{x}) \\
& -\sum_{\operatorname{clr}(\boldsymbol{m}), \boldsymbol{x}} E\{Y \mid Z=1, \operatorname{clr}(\boldsymbol{M})=\operatorname{clr}(\boldsymbol{m}), \boldsymbol{X}=\boldsymbol{x}\} \operatorname{Pr}\{\operatorname{clr}(\boldsymbol{M})=\operatorname{clr}(\boldsymbol{m}) \mid Z=0, \boldsymbol{X}=\boldsymbol{x}\} \operatorname{Pr}(\boldsymbol{X}=\boldsymbol{x}) \\
=& \sum_{\operatorname{clr}(\boldsymbol{m}), \boldsymbol{x}} \Big[E\left\{Y \mid Z=1, \operatorname{clr}(\boldsymbol{M})=\operatorname{clr}(\boldsymbol{m}), \boldsymbol{X}=\boldsymbol{x}\right\} \\
& \quad \times\left\{\operatorname{Pr}\left(\operatorname{clr}(\boldsymbol{M})=\operatorname{clr}(\boldsymbol{m}) \mid Z=1, \boldsymbol{X}=\boldsymbol{x}\right)-\operatorname{Pr}\left(\operatorname{clr}(\boldsymbol{M})=\operatorname{clr}(\boldsymbol{m}) \mid Z=0, \boldsymbol{X}=\boldsymbol{x}\right)\right\} \operatorname{Pr}(\boldsymbol{X}=\boldsymbol{x})\Big].
\end{aligned}
$$

□

S3.2. Proof of Proposition S2

Proof If Assumptions S1–S3 and S4a hold, then

$$
\begin{aligned}
\text{IDE} =& E\left[Y\left\{1, \operatorname{clr}\left(\boldsymbol{G}(0 \mid \boldsymbol{X})\right)\right\}\right] - E\left[Y\left\{0, \operatorname{clr}\left(\boldsymbol{G}(0 \mid \boldsymbol{X})\right)\right\}\right] \\
=& \sum_{\operatorname{clr}(\boldsymbol{m}), \boldsymbol{x}} E[Y\{1, \operatorname{clr}(\boldsymbol{m})\} \mid \operatorname{clr}\{\boldsymbol{G}(0 \mid \boldsymbol{x})\} = \operatorname{clr}(\boldsymbol{m}), \boldsymbol{X} = \boldsymbol{x}] \Pr[\operatorname{clr}\{\boldsymbol{G}(0 \mid \boldsymbol{x})\} = \operatorname{clr}(\boldsymbol{m}) \mid \boldsymbol{X} = \boldsymbol{x}] \Pr(\boldsymbol{X} = \boldsymbol{x}) \\
& - \sum_{\operatorname{clr}(\boldsymbol{m}), \boldsymbol{x}} E[Y\{0, \operatorname{clr}(\boldsymbol{m})\} \mid \operatorname{clr}\{\boldsymbol{G}(0 \mid \boldsymbol{x})\} = \operatorname{clr}(\boldsymbol{m}), \boldsymbol{X} = \boldsymbol{x}] \Pr[\operatorname{clr}\{\boldsymbol{G}(0 \mid \boldsymbol{x})\} = \operatorname{clr}(\boldsymbol{m}) \mid \boldsymbol{X} = \boldsymbol{x}] \Pr(\boldsymbol{X} = \boldsymbol{x}) \\
=& \sum_{\operatorname{clr}(\boldsymbol{m}), \boldsymbol{x}} E[Y\{1, \operatorname{clr}(\boldsymbol{m})\} \mid \boldsymbol{X} = \boldsymbol{x}] \Pr[\operatorname{clr}\{\boldsymbol{M}(0)\} = \operatorname{clr}(\boldsymbol{m}) \mid \boldsymbol{X} = \boldsymbol{x}] \Pr(\boldsymbol{X} = \boldsymbol{x}) \\
& - \sum_{\operatorname{clr}(\boldsymbol{m}), \boldsymbol{x}} E[Y\{0, \operatorname{clr}(\boldsymbol{m})\} \mid \boldsymbol{X} = \boldsymbol{x}] \Pr[\operatorname{clr}\{\boldsymbol{M}(0)\} = \operatorname{clr}(\boldsymbol{m}) \mid \boldsymbol{X} = \boldsymbol{x}] \Pr(\boldsymbol{X} = \boldsymbol{x}) \\
=& \sum_{\operatorname{clr}(\boldsymbol{m}), \boldsymbol{x}} E[Y\{1, \operatorname{clr}(\boldsymbol{m})\} \mid Z = 1, \boldsymbol{X} = \boldsymbol{x}] \Pr[\operatorname{clr}\{\boldsymbol{M}(0)\} = \operatorname{clr}(\boldsymbol{m}) \mid Z = 0, \boldsymbol{X} = \boldsymbol{x}] \Pr(\boldsymbol{X} = \boldsymbol{x}) \\
& - \sum_{\operatorname{clr}(\boldsymbol{m}), \boldsymbol{x}} E[Y\{0, \operatorname{clr}(\boldsymbol{m})\} \mid Z = 0, \boldsymbol{X} = \boldsymbol{x}] \Pr[\operatorname{clr}\{\boldsymbol{M}(0)\} = \operatorname{clr}(\boldsymbol{m}) \mid Z = 0, \boldsymbol{X} = \boldsymbol{x}] \Pr(\boldsymbol{X} = \boldsymbol{x}) \\
=& \sum_{l, \operatorname{clr}(\boldsymbol{m}), \boldsymbol{x}} \Big[E\left\{Y\{1, \operatorname{clr}(\boldsymbol{m})\} \mid Z = 1, L = l, \boldsymbol{X} = \boldsymbol{x}\right\} \Pr(L = l \mid Z = 1, \boldsymbol{X} = \boldsymbol{x}) \\
& \quad \times \Pr[\operatorname{clr}\{\boldsymbol{M}(0)\} = \operatorname{clr}(\boldsymbol{m}) \mid Z = 0, \boldsymbol{X} = \boldsymbol{x}] \Pr(\boldsymbol{X} = \boldsymbol{x})\Big] \\
& - \sum_{l, \operatorname{clr}(\boldsymbol{m}), \boldsymbol{x}} \Big[E\left\{Y\{0, \operatorname{clr}(\boldsymbol{m})\} \mid Z = 0, L = l, \boldsymbol{X} = \boldsymbol{x}\right\} \Pr(L = l \mid Z = 0, \boldsymbol{X} = \boldsymbol{x}) \\
& \quad \times \Pr[\operatorname{clr}\{\boldsymbol{M}(0)\} = \operatorname{clr}(\boldsymbol{m}) \mid Z = 0, \boldsymbol{X} = \boldsymbol{x}] \Pr(\boldsymbol{X} = \boldsymbol{x})\Big] \\
=& \sum_{l, \operatorname{clr}(\boldsymbol{m}), \boldsymbol{x}} \Big[E\left\{Y\{1, \operatorname{clr}(\boldsymbol{m})\} \mid Z = 1, L = l, \operatorname{clr}(\boldsymbol{M}) = \operatorname{clr}(\boldsymbol{m}), \boldsymbol{X} = \boldsymbol{x}\right\} \Pr(L = l \mid Z = 1, \boldsymbol{X} = \boldsymbol{x}) \\
& \quad \times \Pr[\operatorname{clr}\{\boldsymbol{M}(0)\} = \operatorname{clr}(\boldsymbol{m}) \mid Z = 0, \boldsymbol{X} = \boldsymbol{x}] \Pr(\boldsymbol{X} = \boldsymbol{x})\Big] \\
& - \sum_{l, \operatorname{clr}(\boldsymbol{m}), \boldsymbol{x}} \Big[E\left\{Y\{0, \operatorname{clr}(\boldsymbol{m})\} \mid Z = 0, L = l, \operatorname{clr}(\boldsymbol{M}) = \operatorname{clr}(\boldsymbol{m}), \boldsymbol{X} = \boldsymbol{x}\right\} \Pr(L = l \mid Z = 0, \boldsymbol{X} = \boldsymbol{x}) \\
& \quad \times \Pr[\operatorname{clr}\{\boldsymbol{M}(0)\} = \operatorname{clr}(\boldsymbol{m}) \mid Z = 0, \boldsymbol{X} = \boldsymbol{x}] \Pr(\boldsymbol{X} = \boldsymbol{x})\Big] \\
=& \sum_{l, \operatorname{clr}(\boldsymbol{m}), \boldsymbol{x}} \Big[E\{Y \mid Z = 1, L = l, \operatorname{clr}(\boldsymbol{M}) = \operatorname{clr}(\boldsymbol{m}), \boldsymbol{X} = \boldsymbol{x}\} \Pr(L = l \mid Z = 1, \boldsymbol{X} = \boldsymbol{x}) \\
& \quad \times \Pr\{\operatorname{clr}(\boldsymbol{M}) = \operatorname{clr}(\boldsymbol{m}) \mid Z = 0, \boldsymbol{X} = \boldsymbol{x}\} \Pr(\boldsymbol{X} = \boldsymbol{x})\Big] \\
& - \sum_{l, \operatorname{clr}(\boldsymbol{m}), \boldsymbol{x}} \Big[E\{Y \mid Z = 0, L = l, \operatorname{clr}(\boldsymbol{M}) = \operatorname{clr}(\boldsymbol{m}), \boldsymbol{X} = \boldsymbol{x}\} \Pr(L = l \mid Z = 0, \boldsymbol{X} = \boldsymbol{x}) \\
& \quad \times \Pr\{\operatorname{clr}(\boldsymbol{M}) = \operatorname{clr}(\boldsymbol{m}) \mid Z = 0, \boldsymbol{X} = \boldsymbol{x}\} \Pr(\boldsymbol{X} = \boldsymbol{x})\Big] \\
=& \sum_{l, \operatorname{clr}(\boldsymbol{m}), \boldsymbol{x}} \Big[\big\{E(Y \mid Z = 1, L = l, \operatorname{clr}(\boldsymbol{M}) = \operatorname{clr}(\boldsymbol{m}), \boldsymbol{X} = \boldsymbol{x}) \Pr(L = l \mid Z = 1, \boldsymbol{X} = \boldsymbol{x}) \\
& \quad - E\{Y \mid Z = 0, L = l, \operatorname{clr}(\boldsymbol{M}) = \operatorname{clr}(\boldsymbol{m}), \boldsymbol{X} = \boldsymbol{x}\} \Pr(L = l \mid Z = 0, \boldsymbol{X} = \boldsymbol{x})\big\} \\
& \quad \times \Pr\{\operatorname{clr}(\boldsymbol{M}) = \operatorname{clr}(\boldsymbol{m}) \mid Z = 0, \boldsymbol{X} = \boldsymbol{x}\} \Pr(\boldsymbol{X} = \boldsymbol{x})\Big].
\end{aligned}
$$

$$
\begin{aligned}
\mathrm{IIE} =& E\left[Y\left\{1, \mathrm{clr}\left(\boldsymbol{G}(1 \mid \boldsymbol{X})\right)\right\}\right] - E\left[Y\left\{1, \mathrm{clr}\left(\boldsymbol{G}(0 \mid \boldsymbol{X})\right)\right\}\right] \\
=& \sum_{\mathrm{clr}(\boldsymbol{m}),\boldsymbol{x}} E[Y\{1,\mathrm{clr}(\boldsymbol{m})\} \mid \mathrm{clr}\{\boldsymbol{G}(1 \mid x)\} = \mathrm{clr}(\boldsymbol{m}), \boldsymbol{X}=\boldsymbol{x}] \Pr[\mathrm{clr}\{\boldsymbol{G}(1 \mid \boldsymbol{x})\} = \mathrm{clr}(\boldsymbol{m}) \mid \boldsymbol{X}=\boldsymbol{x}] \Pr(\boldsymbol{X}=\boldsymbol{x}) \\
&- \sum_{\mathrm{clr}(\boldsymbol{m}),\boldsymbol{x}} E[Y\{1,\mathrm{clr}(\boldsymbol{m})\} \mid \mathrm{clr}\{\boldsymbol{G}(0 \mid \boldsymbol{x})\} = \mathrm{clr}(\boldsymbol{m}), \boldsymbol{X}=\boldsymbol{x}] \Pr[\mathrm{clr}\{\boldsymbol{G}(0 \mid \boldsymbol{x})\} = \mathrm{clr}(\boldsymbol{m}) \mid \boldsymbol{X}=\boldsymbol{x}] \Pr(\boldsymbol{X}=\boldsymbol{x}) \\
=& \sum_{\mathrm{clr}(\boldsymbol{m}),\boldsymbol{x}} E[Y\{1,\mathrm{clr}(\boldsymbol{m})\} \mid \boldsymbol{X}=\boldsymbol{x}] \Pr[\mathrm{clr}\{\boldsymbol{M}(1)\} = \mathrm{clr}(\boldsymbol{m}) \mid \boldsymbol{X}=\boldsymbol{x}] \Pr(\boldsymbol{X}=\boldsymbol{x}) \\
&- \sum_{\mathrm{clr}(\boldsymbol{m}),\boldsymbol{x}} E[Y\{1,\mathrm{clr}(\boldsymbol{m})\} \mid \boldsymbol{X}=\boldsymbol{x}] \Pr[\mathrm{clr}\{\boldsymbol{M}(0)\} = \mathrm{clr}(\boldsymbol{m}) \mid \boldsymbol{X}=\boldsymbol{x}] \Pr(\boldsymbol{X}=\boldsymbol{x}) \\
=& \sum_{\mathrm{clr}(\boldsymbol{m}),\boldsymbol{x}} E[Y\left\{1,\mathrm{clr}(\boldsymbol{m})\right\} \mid Z=1, \boldsymbol{X}=\boldsymbol{x}] \Pr[\mathrm{clr}\{\boldsymbol{M}(1)\} = \mathrm{clr}(\boldsymbol{m}) \mid Z=1, \boldsymbol{X}=\boldsymbol{x}] \Pr(\boldsymbol{X}=\boldsymbol{x}) \\
&- \sum_{\mathrm{clr}(\boldsymbol{m}),\boldsymbol{x}} E[Y\{1,\mathrm{clr}(\boldsymbol{m})\} \mid Z=1, \boldsymbol{X}=\boldsymbol{x}] \Pr[\mathrm{clr}\{\boldsymbol{M}(0)\} = \mathrm{clr}(\boldsymbol{m}) \mid Z=0, \boldsymbol{X}=\boldsymbol{x}] \Pr(\boldsymbol{X}=\boldsymbol{x}) \\
=& \sum_{l,\mathrm{clr}(\boldsymbol{m}),\boldsymbol{x}} \Big[E\left\{Y\{1,\mathrm{clr}(\boldsymbol{m})\} \mid Z=1, L=l, \boldsymbol{X}=\boldsymbol{x}\right\} \Pr(L=l \mid Z=1, \boldsymbol{X}=\boldsymbol{x}) \\
&\quad \times \Pr[\mathrm{clr}\{\boldsymbol{M}(1)\} = \mathrm{clr}(\boldsymbol{m}) \mid Z=1, \boldsymbol{X}=\boldsymbol{x}] \Pr(\boldsymbol{X}=\boldsymbol{x})\Big] \\
&- \sum_{l,\mathrm{clr}(\boldsymbol{m}),\boldsymbol{x}} \Big[E\left\{Y\left(1,\mathrm{clr}(\boldsymbol{m})\right) \mid Z=1, L=l, \boldsymbol{X}=\boldsymbol{x}\right\} \Pr(L=l \mid Z=1, \boldsymbol{X}=\boldsymbol{x}) \\
&\quad \times \Pr[\mathrm{clr}\{\boldsymbol{M}(0)\} = \mathrm{clr}(\boldsymbol{m}) \mid Z=0, \boldsymbol{X}=\boldsymbol{x}] \Pr(\boldsymbol{X}=\boldsymbol{x})\Big] \\
=& \sum_{l,\mathrm{clr}(\boldsymbol{m}),\boldsymbol{x}} \Big[E\left\{Y\left(1,\mathrm{clr}(\boldsymbol{m})\right) \mid Z=1, L=l, \mathrm{clr}(\boldsymbol{M}) = \mathrm{clr}(\boldsymbol{m}), \boldsymbol{X}=\boldsymbol{x}\right\} \Pr(L=l \mid Z=1, \boldsymbol{X}=\boldsymbol{x}) \\
&\quad \times \Pr[\mathrm{clr}\{\boldsymbol{M}(1)\} = \mathrm{clr}(\boldsymbol{m}) \mid Z=1, \boldsymbol{X}=\boldsymbol{x}] \Pr(\boldsymbol{X}=\boldsymbol{x})\Big] \\
&- \sum_{l,\mathrm{clr}(\boldsymbol{m}),\boldsymbol{x}} \Big[E\Big\{Y\left(1,\mathrm{clr}(\boldsymbol{m})\right) \mid Z=1, L=l, \mathrm{clr}(\boldsymbol{M}) = \mathrm{clr}(\boldsymbol{m}), \boldsymbol{X}=\boldsymbol{x}\Big\} \Pr(L=l \mid Z=1, \boldsymbol{X}=\boldsymbol{x}) \\
&\quad \times \Pr[\mathrm{clr}\{\boldsymbol{M}(0)\} = \mathrm{clr}(\boldsymbol{m}) \mid Z=0, \boldsymbol{X}=\boldsymbol{x}] \Pr(\boldsymbol{X}=\boldsymbol{x})\Big] \\
=& \sum_{l,\mathrm{clr}(\boldsymbol{m}),\boldsymbol{x}} \Big[E\{Y \mid Z=1, L=l, \mathrm{clr}(\boldsymbol{M}) = \mathrm{clr}(\boldsymbol{m}), \boldsymbol{X}=\boldsymbol{x}\} \Pr(L=l \mid Z=1, \boldsymbol{X}=\boldsymbol{x}) \\
&\quad \times \Pr\{\mathrm{clr}(\boldsymbol{M}) = \mathrm{clr}(\boldsymbol{m}) \mid Z=1, \boldsymbol{X}=\boldsymbol{x}\} \Pr(\boldsymbol{X}=\boldsymbol{x})\Big] \\
&- \sum_{l,\mathrm{clr}(\boldsymbol{m}),\boldsymbol{x}} \Big[E\{Y \mid Z=1, L=l, \mathrm{clr}(\boldsymbol{M}) = \mathrm{clr}(\boldsymbol{m}), \boldsymbol{X}=\boldsymbol{x}\} \Pr(L=l \mid Z=1, \boldsymbol{X}=\boldsymbol{x}) \\
&\quad \times \Pr\{\mathrm{clr}(\boldsymbol{M}) = \mathrm{clr}(\boldsymbol{m}) \mid Z=0, \boldsymbol{X}=\boldsymbol{x}\} \Pr(\boldsymbol{X}=\boldsymbol{x})\Big] \\
=& \sum_{l,\mathrm{clr}(\boldsymbol{m}),\boldsymbol{x}} \Big[E\left\{Y \mid Z=1, L=l, \mathrm{clr}(\boldsymbol{M}) = \mathrm{clr}(\boldsymbol{m}), \boldsymbol{X}=\boldsymbol{x}\right\} \Pr(L=l \mid Z=1, \boldsymbol{X}=\boldsymbol{x}) \\
&\times \left\{\Pr\left(\mathrm{clr}(\boldsymbol{M}) = \mathrm{clr}(\boldsymbol{m}) \mid Z=1, \boldsymbol{X}=\boldsymbol{x}\right) - \Pr\left(\mathrm{clr}(\boldsymbol{M}) = \mathrm{clr}(\boldsymbol{m}) \mid Z=0, \boldsymbol{X}=\boldsymbol{x}\right)\right\} \Pr(\boldsymbol{X}=\boldsymbol{x})\Big].
\end{aligned}
$$

□

S3.3. Proof of Theorem 1 in the main manuscript

Proof Under Assumptions S1–S3, S4a, and S6, Proposition S2 gives

$$
\begin{aligned}
\mathrm{IDE} &= \sum_{l,\mathrm{clr}(\boldsymbol{m}),\boldsymbol{x}} \left[E\{Y \mid z_1, l, \mathrm{clr}(\boldsymbol{m}), \boldsymbol{x}\} P(l \mid z_1, \boldsymbol{x}) - E\{Y \mid z_0, l, \mathrm{clr}(\boldsymbol{m}), \boldsymbol{x}\} P(l \mid z_0, \boldsymbol{x})\right] P\{\mathrm{clr}(\boldsymbol{m}) \mid z_0, \boldsymbol{x}\} P(\boldsymbol{x}); \\
\mathrm{IIE} &= \sum_{l,\mathrm{clr}(\boldsymbol{m}),\boldsymbol{x}} E\{Y \mid z_1, l, \mathrm{clr}(\boldsymbol{m}), \boldsymbol{x}\} P(l \mid z_1, \boldsymbol{x}) \left[P\{\mathrm{clr}(\boldsymbol{m}) \mid z_1, \boldsymbol{x}\} - P\{\mathrm{clr}(\boldsymbol{m}) \mid z_0, \boldsymbol{x}\}\right] P(\boldsymbol{x}).
\end{aligned}
\tag{S3}
$$

Let

$$\eta_1 = \sum_{l,\mathrm{clr}(\boldsymbol{m}),\boldsymbol{x}} E\{Y \mid z_1, l, \mathrm{clr}(\boldsymbol{m}), \boldsymbol{x}\} P(l \mid z_1, \boldsymbol{x}) P\{\mathrm{clr}(\boldsymbol{m}) \mid z_0, \boldsymbol{x}\} P(\boldsymbol{x});$$

$$\eta_2 = \sum_{l,\mathrm{clr}(\boldsymbol{m}),\boldsymbol{x}} E\{Y \mid z_0, l, \mathrm{clr}(\boldsymbol{m}), \boldsymbol{x}\} P(l \mid z_0, \boldsymbol{x}) P\{\mathrm{clr}(\boldsymbol{m}) \mid z_0, \boldsymbol{x}\} P(\boldsymbol{x});$$

$$\eta_3 = \sum_{l,\mathrm{clr}(\boldsymbol{m}),\boldsymbol{x}} E\{Y \mid z_1, l, \mathrm{clr}(\boldsymbol{m}), \boldsymbol{x}\} P(l \mid z_1, \boldsymbol{x}) P\{\mathrm{clr}(\boldsymbol{m}) \mid z_1, \boldsymbol{x}\} P(\boldsymbol{x}).$$

Assumption S6 ensures that the denominators in the following weighting representations are positive on the relevant support.
First, we show that

$$\eta_1 = E\left[\frac{\mathbb{I}(Z=z_0)Y}{\Pr(Z=z_0 \mid \boldsymbol{X})} \frac{E\{Y \mid Z=z_1, L, \mathrm{clr}(\boldsymbol{M}), \boldsymbol{X}\} \Pr(L \mid Z=z_1, \boldsymbol{X})}{E\{Y \mid Z=z_0, L, \mathrm{clr}(\boldsymbol{M}), \boldsymbol{X}\} \Pr\{L \mid Z=z_0, \mathrm{clr}(\boldsymbol{M}), \boldsymbol{X}\}}\right]. \tag{S4}$$

$$\begin{aligned}
&\text{RHS of (S4)} \\
=&E_{Z,L,\mathrm{clr}(\boldsymbol{M}),\boldsymbol{X}} E\left[\frac{\mathbb{I}(Z=z_0)Y}{\Pr(Z=z_0 \mid \boldsymbol{X})} \frac{E\{Y \mid Z=z_1, L, \mathrm{clr}(\boldsymbol{M}), \boldsymbol{X}\} \Pr(L \mid Z=z_1, \boldsymbol{X})}{E\{Y \mid Z=z_0, L, \mathrm{clr}(\boldsymbol{M}), \boldsymbol{X}\} \Pr\{L \mid Z=z_0, \mathrm{clr}(\boldsymbol{M}), \boldsymbol{X}\}} \middle| Z, L, \mathrm{clr}(\boldsymbol{M}), \boldsymbol{X}\right] \\
=&\sum_{l,\mathrm{clr}(\boldsymbol{m}),\boldsymbol{x}} \left[E\left\{\frac{Y}{\Pr(Z=z_0 \mid \boldsymbol{X}=\boldsymbol{x})} \frac{E\{Y \mid z_1, l, \mathrm{clr}(\boldsymbol{m}), \boldsymbol{x}\} P(l \mid z_1, \boldsymbol{x})}{E\{Y \mid z_0, l, \mathrm{clr}(\boldsymbol{m}), \boldsymbol{x}\} P\{l \mid z_0, \mathrm{clr}(\boldsymbol{m}), \boldsymbol{x}\}} \middle| Z=z_0, l, \mathrm{clr}(\boldsymbol{m}), \boldsymbol{x}\right\}\right. \\
&\qquad \left.\times P(\boldsymbol{x}) P(z_0 \mid \boldsymbol{x}) P\{l, \mathrm{clr}(\boldsymbol{m}) \mid z_0, \boldsymbol{x}\}\right] \\
=&\sum_{l,\mathrm{clr}(\boldsymbol{m}),\boldsymbol{x}} \frac{E\{Y \mid z_0, l, \mathrm{clr}(\boldsymbol{m}), \boldsymbol{x}\}}{\Pr(Z=z_0 \mid \boldsymbol{X}=\boldsymbol{x})} \frac{E\{Y \mid z_1, l, \mathrm{clr}(\boldsymbol{m}), \boldsymbol{x}\} P(l \mid z_1, \boldsymbol{x})}{E\{Y \mid z_0, l, \mathrm{clr}(\boldsymbol{m}), \boldsymbol{x}\} P\{l \mid z_0, \mathrm{clr}(\boldsymbol{m}), \boldsymbol{x}\}} P(\boldsymbol{x}) P(z_0 \mid \boldsymbol{x}) P\{l, \mathrm{clr}(\boldsymbol{m}) \mid z_0, \boldsymbol{x}\} \\
=&\sum_{l,\mathrm{clr}(\boldsymbol{m}),\boldsymbol{x}} E\{Y \mid z_1, l, \mathrm{clr}(\boldsymbol{m}), \boldsymbol{x}\} P(l \mid z_1, \boldsymbol{x}) P\{\mathrm{clr}(\boldsymbol{m}) \mid z_0, \boldsymbol{x}\} P(\boldsymbol{x}) \\
=&\text{LHS of (S4)}.
\end{aligned}$$

Second, we show that

$$\eta_2 = E\left[\frac{\mathbb{I}(Z=z_0)Y}{\Pr(Z=z_0 \mid \boldsymbol{X})} \frac{E\{Y \mid Z=z_0, L, \mathrm{clr}(\boldsymbol{M}), \boldsymbol{X}\} \Pr(L \mid Z=z_0, \boldsymbol{X})}{E\{Y \mid Z=z_0, L, \mathrm{clr}(\boldsymbol{M}), \boldsymbol{X}\} \Pr\{L \mid Z=z_0, \mathrm{clr}(\boldsymbol{M}), \boldsymbol{X}\}}\right]. \tag{S5}$$

$$\begin{aligned}
&\text{RHS of (S5)} \\
=&E_{Z,L,\mathrm{clr}(\boldsymbol{M}),\boldsymbol{X}} E\left[\frac{\mathbb{I}(Z=z_0)Y}{\Pr(Z=z_0 \mid \boldsymbol{X})} \frac{E\{Y \mid Z=z_0, L, \mathrm{clr}(\boldsymbol{M}), \boldsymbol{X}\} \Pr(L \mid Z=z_0, \boldsymbol{X})}{E\{Y \mid Z=z_0, L, \mathrm{clr}(\boldsymbol{M}), \boldsymbol{X}\} \Pr\{L \mid Z=z_0, \mathrm{clr}(\boldsymbol{M}), \boldsymbol{X}\}} \middle| Z, L, \mathrm{clr}(\boldsymbol{M}), \boldsymbol{X}\right] \\
=&\sum_{l,\mathrm{clr}(\boldsymbol{m}),\boldsymbol{x}} \left[E\left\{\frac{Y}{\Pr(Z=z_0 \mid \boldsymbol{X}=\boldsymbol{x})} \frac{E\{Y \mid z_0, l, \mathrm{clr}(\boldsymbol{m}), \boldsymbol{x}\} P(l \mid z_0, \boldsymbol{x})}{E\{Y \mid z_0, l, \mathrm{clr}(\boldsymbol{m}), \boldsymbol{x}\} P\{l \mid z_0, \mathrm{clr}(\boldsymbol{m}), \boldsymbol{x}\}} \middle| \boldsymbol{x}, Z=z_0, l, \mathrm{clr}(\boldsymbol{m})\right\}\right. \\
&\qquad \left.\times P(\boldsymbol{x}) P(z_0 \mid \boldsymbol{x}) P\{l, \mathrm{clr}(\boldsymbol{m}) \mid z_0, \boldsymbol{x}\}\right] \\
=&\sum_{l,\mathrm{clr}(\boldsymbol{m}),\boldsymbol{x}} \frac{E\{Y \mid z_0, l, \mathrm{clr}(\boldsymbol{m}), \boldsymbol{x}\}}{\Pr(Z=z_0 \mid \boldsymbol{X}=\boldsymbol{x})} \frac{E\{Y \mid z_0, l, \mathrm{clr}(\boldsymbol{m}), \boldsymbol{x}\} P(l \mid z_0, \boldsymbol{x})}{E\{Y \mid z_0, l, \mathrm{clr}(\boldsymbol{m}), \boldsymbol{x}\} P(l \mid z_0, \mathrm{clr}(\boldsymbol{m}), \boldsymbol{x})} P(\boldsymbol{x}) P(z_0 \mid \boldsymbol{x}) P\{l, \mathrm{clr}(\boldsymbol{m}) \mid z_0, \boldsymbol{x}\} \\
=&\sum_{l,\mathrm{clr}(\boldsymbol{m}),\boldsymbol{x}} E\{Y \mid z_0, l, \mathrm{clr}(\boldsymbol{m}), \boldsymbol{x}\} P(l \mid z_0, \boldsymbol{x}) P\{\mathrm{clr}(\boldsymbol{m}) \mid z_0, \boldsymbol{x}\} P(\boldsymbol{x}) \\
=&\text{LHS of (S5)}.
\end{aligned}$$

Third, we prove that

$$\eta_3 = E\left[\frac{\mathbb{I}(Z=z_1)Y}{\Pr(Z=z_1 \mid \boldsymbol{X})} \frac{\Pr(L \mid Z=z_1, \boldsymbol{X})}{\Pr\{L \mid Z=z_1, \mathrm{clr}(\boldsymbol{M}), \boldsymbol{X}\}}\right]. \tag{S6}$$

RHS of (S6)

$$= E_{Z,L,\mathrm{clr}(\boldsymbol{M}),\boldsymbol{X}} E\left[\frac{\mathbb{I}(Z=z_1)Y}{\Pr(Z=z_1\mid \boldsymbol{X})}\frac{\Pr(L\mid Z=z_1,\boldsymbol{X})}{\Pr\{L\mid Z=z_1,\mathrm{clr}(\boldsymbol{M}),\boldsymbol{X}\}}\middle| Z,L,\mathrm{clr}(\boldsymbol{M}),\boldsymbol{X}\right]$$

$$= \sum_{l,\mathrm{clr}(\boldsymbol{m}),\boldsymbol{x}} \frac{E\{Y\mid z_1,l,\mathrm{clr}(\boldsymbol{m}),\boldsymbol{x}\}}{P(z_1\mid \boldsymbol{x})}\frac{P(l\mid z_1,\boldsymbol{x})}{P\{l\mid z_1,\mathrm{clr}(\boldsymbol{m}),\boldsymbol{x}\}}P(\boldsymbol{x})P(z_1\mid \boldsymbol{x})P\{l,\mathrm{clr}(\boldsymbol{m})\mid z_1,\boldsymbol{x}\}$$

$$= \sum_{l,\mathrm{clr}(\boldsymbol{m}),\boldsymbol{x}} E\{Y\mid z_1,l,\mathrm{clr}(\boldsymbol{m}),\boldsymbol{x}\}P(l\mid z_1,\boldsymbol{x})\frac{P\{l\mid z_1,\mathrm{clr}(\boldsymbol{m}),\boldsymbol{x}\}P\{\mathrm{clr}(\boldsymbol{m})\mid z_1,\boldsymbol{x}\}}{P\{l\mid z_1,\mathrm{clr}(\boldsymbol{m}),\boldsymbol{x}\}}P(\boldsymbol{x})$$

$$= \sum_{l,\mathrm{clr}(\boldsymbol{m}),\boldsymbol{x}} E\{Y\mid z_1,l,\mathrm{clr}(\boldsymbol{m}),\boldsymbol{x}\}P(l\mid z_1,\boldsymbol{x})P\{\mathrm{clr}(\boldsymbol{m})\mid z_1,\boldsymbol{x}\}P(\boldsymbol{x})$$

= LHS of (S6).

Therefore, based on (S3),

$$\begin{aligned}\mathrm{IDE} =&\eta_1-\eta_2\\ =&E\left[\frac{\mathbb{I}(Z=z_0)Y}{\Pr(Z=z_0\mid \boldsymbol{X})}\frac{E\{Y\mid Z=z_1,L,\mathrm{clr}(\boldsymbol{M}),\boldsymbol{X}\}\Pr(L\mid Z=z_1,\boldsymbol{X})}{E\{Y\mid Z=z_0,L,\mathrm{clr}(\boldsymbol{M}),\boldsymbol{X}\}\Pr\{L\mid Z=z_0,\mathrm{clr}(\boldsymbol{M}),\boldsymbol{X}\}}\right]\\ &-E\left[\frac{\mathbb{I}(Z=z_0)Y}{\Pr(Z=z_0\mid \boldsymbol{X})}\frac{\Pr(L\mid Z=z_0,\boldsymbol{X})}{\Pr\{L\mid Z=z_0,\mathrm{clr}(\boldsymbol{M}),\boldsymbol{X}\}}\right];\end{aligned}$$

$$\begin{aligned}\mathrm{IIE} =&\eta_3-\eta_1\\ =&E\left[\frac{\mathbb{I}(Z=z_1)Y}{\Pr(Z=z_1\mid \boldsymbol{X})}\frac{\Pr(L\mid Z=z_1,\boldsymbol{X})}{\Pr\{L\mid Z=z_1,\mathrm{clr}(\boldsymbol{M}),\boldsymbol{X}\}}\right]\\ &-E\left[\frac{\mathbb{I}(Z=z_0)Y}{\Pr(Z=z_0\mid \boldsymbol{X})}\frac{E\{Y\mid Z=z_1,L,\mathrm{clr}(\boldsymbol{M}),\boldsymbol{X}\}\Pr(L\mid Z=z_1,\boldsymbol{X})}{E\{Y\mid Z=z_0,L,\mathrm{clr}(\boldsymbol{M}),\boldsymbol{X}\}\Pr\{L\mid Z=z_0,\mathrm{clr}(\boldsymbol{M}),\boldsymbol{X}\}}\right].\end{aligned}$$

□

S3.4. Proof of Corollary 1 in the main manuscript

Proof Under the conditional independence assumption in Corollary 1,

$$Y \perp\!\!\!\perp \mathrm{clr}(\boldsymbol{M}^{(2)})\mid \{Z,L,\boldsymbol{X},\mathrm{clr}(\boldsymbol{M}^{(1)})\},$$

we have

$$E\{Y\mid Z,L,\mathrm{clr}(\boldsymbol{M}),\boldsymbol{X}\}=E\{Y\mid Z,L,\mathrm{clr}(\boldsymbol{M}^{(1)}),\boldsymbol{X}\}.$$

Substituting this equality into the identification formulas in Proposition S2 and summing over $\mathrm{clr}(\boldsymbol{m}^{(2)})$ yields

$$\mathrm{IDE}=\sum_{l,\mathrm{clr}(\boldsymbol{m}^{(1)}),\boldsymbol{x}}\left[\left\{E\{Y\mid z_1,l,\mathrm{clr}(\boldsymbol{m}^{(1)}),\boldsymbol{x}\}P(l\mid z_1,\boldsymbol{x})-E\{Y\mid z_0,l,\mathrm{clr}(\boldsymbol{m}^{(1)}),\boldsymbol{x}\}P(l\mid z_0,\boldsymbol{x})\right\}P\{\mathrm{clr}(\boldsymbol{m}^{(1)})\mid z_0,\boldsymbol{x}\}P(\boldsymbol{x})\right];$$

$$\mathrm{IIE}=\sum_{l,\mathrm{clr}(\boldsymbol{m}^{(1)}),\boldsymbol{x}}E\{Y\mid z_1,l,\mathrm{clr}(\boldsymbol{m}^{(1)}),\boldsymbol{x}\}P(l\mid z_1,\boldsymbol{x})\Big[P\{\mathrm{clr}(\boldsymbol{m}^{(1)})\mid z_1,\boldsymbol{x}\}-P\{\mathrm{clr}(\boldsymbol{m}^{(1)})\mid z_0,\boldsymbol{x}\}\Big]P(\boldsymbol{x}).$$

Here, summations are replaced by integrals as appropriate for continuous variables. The marginalization step follows from

$$\sum_{\mathrm{clr}(\boldsymbol{m}^{(2)})}P\{\mathrm{clr}(\boldsymbol{m}^{(1)}),\mathrm{clr}(\boldsymbol{m}^{(2)})\mid z,\boldsymbol{x}\}=P\{\mathrm{clr}(\boldsymbol{m}^{(1)})\mid z,\boldsymbol{x}\}.$$

Under Assumption S6, the denominators in the following weighting representations are positive on the relevant support. Applying the same iterated-expectation calculations as in the proof of Theorem 1 in Section S3.3, with $\mathrm{clr}(\boldsymbol{M})$ replaced by $\mathrm{clr}(\boldsymbol{M}^{(1)})$, gives

$$\begin{aligned}\mathrm{IDE}=&E\left[\frac{\mathbb{I}(Z=z_0)Y}{\Pr(Z=z_0\mid \boldsymbol{X})}\frac{E\{Y\mid Z=z_1,L,\mathrm{clr}(\boldsymbol{M}^{(1)}),\boldsymbol{X}\}}{E\{Y\mid Z=z_0,L,\mathrm{clr}(\boldsymbol{M}^{(1)}),\boldsymbol{X}\}}\frac{\Pr(L\mid Z=z_1,\boldsymbol{X})}{\Pr\{L\mid Z=z_0,\mathrm{clr}(\boldsymbol{M}^{(1)}),\boldsymbol{X}\}}\right]\\ &-E\left[\frac{\mathbb{I}(Z=z_0)Y}{\Pr(Z=z_0\mid \boldsymbol{X})}\frac{\Pr(L\mid Z=z_0,\boldsymbol{X})}{\Pr\{L\mid Z=z_0,\mathrm{clr}(\boldsymbol{M}^{(1)}),\boldsymbol{X}\}}\right];\end{aligned}$$

and

$$\text{IIE} = E\left[\frac{\mathbb{I}(Z=z_1)Y}{\Pr(Z=z_1 \mid \boldsymbol{X})}\frac{\Pr(L \mid Z=z_1,\boldsymbol{X})}{\Pr\{L \mid Z=z_1,\text{clr}(\boldsymbol{M}^{(1)}),\boldsymbol{X}\}}\right]$$

$$- E\left[\frac{\mathbb{I}(Z=z_0)Y}{\Pr(Z=z_0 \mid \boldsymbol{X})}\frac{E\{Y \mid Z=z_1,L,\text{clr}(\boldsymbol{M}^{(1)}),\boldsymbol{X}\}}{E\{Y \mid Z=z_0,L,\text{clr}(\boldsymbol{M}^{(1)}),\boldsymbol{X}\}}\frac{\Pr(L \mid Z=z_1,\boldsymbol{X})}{\Pr\{L \mid Z=z_0,\text{clr}(\boldsymbol{M}^{(1)}),\boldsymbol{X}\}}\right].$$

Since Z is binary with $z_0 = 0$ and $z_1 = 1$, $\mathbb{I}(Z = z_0) = 1 - Z$ and $\mathbb{I}(Z = z_1) = Z$. These expressions therefore coincide with those in Corollary 1.
□

Supplementary References

F. D'Amico, A. M. Perrone, S. Rampelli, S. Coluccelli, M. Barone, G. Ravegnini, M. Fabbrini, P. Brigidi, P. De Iaco, and S. Turroni. Gut microbiota dynamics during chemotherapy in epithelial ovarian cancer patients are related to therapeutic outcome. *Cancers*, 13(16):3999, 2021.

B. Efron. Better bootstrap confidence intervals. *Journal of the American statistical Association*, 82(397):171–185, 1987.

B. Efron and R. J. Tibshirani. *An introduction to the bootstrap*. CRC press, 1994.

H. Hakim, R. Dallas, J. Wolf, L. Tang, S. Schultz-Cherry, V. Darling, C. Johnson, E. A. Karlsson, T.-C. Chang, S. Jeha, et al. Gut microbiome composition predicts infection risk during chemotherapy in children with acute lymphoblastic leukemia. *Clinical Infectious Diseases*, 67(4):541–548, 2018.

S. Khanna, E. Montassier, B. Schmidt, R. Patel, D. Knights, D. S. Pardi, and P. Kashyap. Gut microbiome predictors of treatment response and recurrence in primary clostridium difficile infection. *Alimentary pharmacology & therapeutics*, 44(7):715–727, 2016.

D. P. Kingma and J. Ba. Adam: A method for stochastic optimization. *arXiv preprint arXiv:1412.6980*, 2014.

H. Lin and S. D. Peddada. Analysis of microbial compositions: a review of normalization and differential abundance analysis. *NPJ biofilms and microbiomes*, 6(1):60, 2020.

S. McMahon, S. Franklin, and J. Galloway-Peña. Utilization of machine learning to predict antibiotic resistant event outcomes in acute myeloid leukemia patients undergoing induction chemotherapy. *Frontiers in Cellular and Infection Microbiology*, 15:1629422, 2025.

X. Mi, F. Zou, and R. Zhu. Bagging and deep learning in optimal individualized treatment rules. *Biometrics*, 75(2):674–684, 2019.

L. E. Papanicolas, S. K. Sims, S. L. Taylor, S. J. Miller, C. S. Karapetis, S. L. Wesselingh, D. L. Gordon, and G. B. Rogers. Conventional myelosuppressive chemotherapy for non-haematological malignancy disrupts the intestinal microbiome. *BMC cancer*, 21(1):591, 2021.

J. Pearl. Direct and indirect effects. In *Proceedings of the Seventeenth conference on Uncertainty in artificial intelligence*, pages 411–420, 2001.

J. M. Robins and T. S. Richardson. Alternative graphical causal models and the identification of direct effects. *Causality and psychopathology: Finding the determinants of disorders and their cures*, pages 103–158, 2010.

W. Sangster, J. P. Hegarty, K. M. Schieffer, J. R. Wright, J. Hackman, D. R. Toole, R. Lamendella, and D. B. Stewart Sr. Bacterial and fungal microbiota changes distinguish c. difficile infection from other forms of diarrhea: results of a prospective inpatient study. *Frontiers in microbiology*, 7:789, 2016.

S. Terrisse, L. Derosa, V. Iebba, F. Ghiringhelli, I. Vaz-Luis, G. Kroemer, M. Fidelle, S. Christodoulidis, N. Segata, A. M. Thomas, et al. Intestinal microbiota influences clinical outcome and side effects of early breast cancer treatment. *Cell Death & Differentiation*, 28(9):2778–2796, 2021.

T. J. VanderWeele, S. Vansteelandt, and J. M. Robins. Effect decomposition in the presence of an exposure-induced mediator-outcome confounder. *Epidemiology*, 25(2):300, 2014.

S. Vázquez-Cuesta, L. Villar, N. L. García, A. I. Fernández, M. Olmedo, L. Alcalá, M. Marín, P. Muñoz, E. Bouza, and E. Reigadas. Characterization of the gut microbiome of patients with clostridioides difficile infection, patients with non–c. difficile diarrhea, and c. difficile–colonized patients. *Frontiers in cellular and infection microbiology*, 13:1130701, 2023.

C. Vincent, D. A. Stephens, V. G. Loo, T. J. Edens, M. A. Behr, K. Dewar, and A. R. Manges. Reductions in intestinal clostridiales precede the development of nosocomial clostridium difficile infection. *Microbiome*, 1(1):18, 2013.

A. H. Wu, C. Vigen, C. Tseng, A. A. Garcia, and D. Spicer. Effect of chemotherapy on the gut microbiome of breast cancer patients during the first year of treatment. *Breast Cancer: Targets and Therapy*, pages 433–451, 2022.

Z.-F. Xu, L. Yuan, Y. Zhang, W. Zhang, C. Wei, W. Wang, D. Zhao, D. Zhou, and J. Li. The gut microbiome correlated to chemotherapy efficacy in diffuse large b-cell lymphoma patients. *Hematology Reports*, 16(1):63–75, 2024.

M. Zhang, D. Liu, H. Zhou, X. Liu, X. Li, Y. Cheng, B. Gao, and J. Chen. Intestinal flora characteristics of advanced non-small cell lung cancer in china and their role in chemotherapy based on metagenomics: A prospective exploratory cohort study. *Thoracic cancer*, 12(24):3293–3303, 2021.
